# Few-shot out-of-distribution detection for automated screening in retinal OCT images using deep learning

**DOI:** 10.1038/s41598-023-43018-9

**Published:** 2023-09-27

**Authors:** Teresa Araújo, Guilherme Aresta, Ursula Schmidt-Erfurth, Hrvoje Bogunović

**Affiliations:** Christian Doppler Laboratory for Artificial Intelligence in Retina, Department of Ophthalmology and Optometry, Medical University of Vienna, Vienna, Austria

**Keywords:** Computer science, Eye diseases, Biomedical engineering, Medical imaging

## Abstract

Deep neural networks have been increasingly proposed for automated screening and diagnosis of retinal diseases from optical coherence tomography (OCT), but often provide high-confidence predictions on out-of-distribution (OOD) cases, compromising their clinical usage. With this in mind, we performed an in-depth comparative analysis of the state-of-the-art uncertainty estimation methods for OOD detection in retinal OCT imaging. The analysis was performed within the use-case of automated screening and staging of age-related macular degeneration (AMD), one of the leading causes of blindness worldwide, where we achieved a macro-average area under the curve (AUC) of 0.981 for AMD classification. We focus on a few-shot Outlier Exposure (OE) method and the detection of near-OOD cases that share pathomorphological characteristics with the inlier AMD classes. Scoring the OOD case based on the Cosine distance in the feature space from the penultimate network layer proved to be a robust approach for OOD detection, especially in combination with the OE. Using Cosine distance and only 8 outliers exposed per class, we were able to improve the near-OOD detection performance of the OE with Reject Bucket method by $\approx$ 10% compared to without OE, reaching an AUC of 0.937. The Cosine distance served as a robust metric for OOD detection of both known and unknown classes and should thus be considered as an alternative to the reject bucket class probability in OE approaches, especially in the few-shot scenario. The inclusion of these methodologies did not come at the expense of classification performance, and can substantially improve the reliability and trustworthiness of the resulting deep learning-based diagnostic systems in the context of retinal OCT.

## Introduction

Optical coherence tomography (OCT)^1^ has become the gold standard imaging technique for the diagnosis and management of retinal diseases, having gained popularity over other imaging techniques, such as color fundus photography^2^. It is non-invasive and allows acquiring a dense three-dimensional cross-sectional scan of the retina, with micrometer resolution, in a matter of seconds. One clinical setting in which OCT imaging is of particular value is in age-related macular degeneration (AMD) diagnosis and staging^2^.

**Figure 1:**
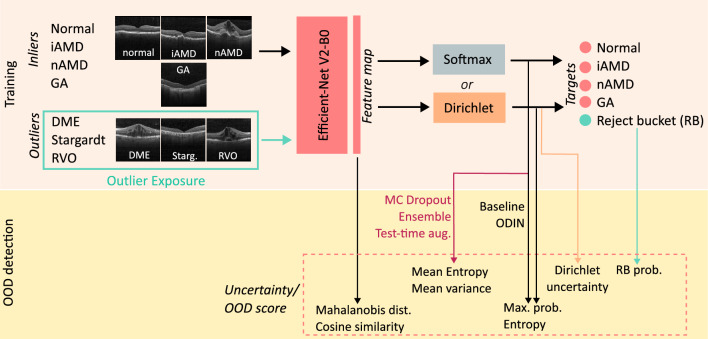
Workflow of the proposed method for outlier class detection.

AMD is a leading cause of blindness globally^3,4^, and thus its automated detection and staging is of paramount importance. AMD in OCT images can be staged based on different clinical manifestations. Early and intermediate AMD (iAMD) are characterized by the deposition of drusen between the retinal pigment epithelium (RPE) and the Bruch’s membrane^5^. iAMD commonly progresses to one of two late stages, neovascular or wet AMD (nAMD), with growth of abnormal vessels most commonly from the choroid, through macular neovascularization^6^, or geographical atrophy (GA), corresponding to the death of RPE cells, photoreceptors, and/or choriocapillaris^7^. In addition to AMD, OCT is used for the diagnosis of other diseases such as diabetic macular edema (DME), retinal vein occlusion (RVO) or Stargardt disease. Both DME and RVO are mainly characterized by the accumulation of intra- or subretinal fluid^8,9^. Interestingly, the signs of these two pathologies are very similar in OCT images, leading ophtalmologists to use fundus photography to facilitate differentiating between them^10^. On the other hand, Stargardt, an inherited retinal disorder, manifests similarly to GA, though without the presence of drusen^11,12^. Representative examples of these pathologies are shown in Fig. 1.

Despite the great applicability of OCT for retinal disease diagnosis, particularly in AMD screening, manual inspection and qualitative analysis of the dense OCT volumes is time-consuming and tiresome for retinal specialists^13^, increasing the risk of misdiagnosis. Convolutional neural networks (CNNs) have shown a large potential for automated retinal disease screening with OCT due to their strong image recognition capabilities^14–17^. However, a caveat of training CNNs, of special concern in the medical field, is that these systems often fail to generalize to pathologies other than those provided during training (*inliers*), inferring overconfident predictions for out-of-distribution (OOD) cases (*outliers*). This severely impacts the clinical safety of deep learning systems for (semi-)automated diagnosis, limiting their trustworthiness and applicability in real-world scenarios.

Over the past years, a large effort has been made to improve the reliability of systems by developing techniques for uncertainty estimation and automated OOD detection^18–21^. However, the vast majority of the approaches focuses on far-OOD in contrast to near-OOD, which are much more challenging to detect due to their similarity to the in-distribution data^22^. A near-OOD would be, e.g. an OCT volume with similar acquisition settings to the training set but of an unseen pathology, and thus their detection is highly relevant in a clinical setting. However, most of the existing approaches tend to fail for near-OOD cases^23–25^. A possible solution is to train CNNs using a set of representative OOD cases, a technique known as *outlier exposure* (OE). Recently, it has been shown for dermatological images that OE with a large number of severely under-represented classes improves the models’ overall OOD detection performance for outlier classes other than those seen during training^26^.

The high acquisition and labeling costs of retinal OCT, in contrast to other modalities such as photography in dermatology, limit the availability of samples for OE. Because of this, a large-scale OE approach is not easily employable for this imaging modality. Instead, it is of interest to understand the number of exposed outliers needed to increase the robustness of the system, i.e., understand the behavior of a retinal OCT screening model under a few-shot outlier exposure scenario. Furthermore, as suggested by recent studies, OOD detection methods can yield unexpected results on near-OOD cases^27,28^, and thus the thorough analysis of these methods within a concrete medical context is of paramount interest.

### Related work

The vast majority of state-of-the-art methods for the classification of the aforementioned diseases are deep learning-based, with research focusing on a binary^29^ or multiclass classification problems. For example, fine-tuned models have been successfully applied for 3-class^30–32^ and 4-class^15,33–36^ classification. These approaches used a single (usually central) B-scan collected from the OCT volume, but other authors also considered 3D models to improve the classification performance^14,37–39^. In general, the majority of OCT classification methods present a very high classification performance, with accuracies ranging from 85% up to 99%. However, most studies require a large number of training images (e.g. the dataset used in Kermany *et al.*^15^ has more than 100,000 images), do not perform an inter-dataset assessment, and/or consider images acquired by a device from a single manufacturer^20^ (Supplementary material, Sec. 1.1, provides an overview of the characteristics of the datasets used in different OCT classification studies).

Despite their high classification performance, deep learning classification models are still not used in clinical practice. In particular, their black-box behaviour and overconfident predictions even on complex cases or those outside the training domain undermine the trust of their users^40,41^. OOD detection, which is pivotal for easing the translation and adoption of these systems in the real-world, is usually performed either by directly predicting if a sample is an outlier or by making a decision over a prediction uncertainty estimation. Uncertainty estimation in medical imaging, and in particular in ophthalmology, is usually embedded during training by modeling the classification as a distribution or posteriorly via an ensemble of predictions. For instance, Gaussian- and Dirichlet-based modeling has successfully been used to improve the reliability in the classification of eye fundus images^42,43^. Post-training uncertainty estimation is also very common as it can be performed without changing the architecture of the network. A notable approach is uncertainty estimation via Monte Carlo (MC) dropout^18^, which has been applied for referable DR detection in eye fundus images^44^ as well as in OCT classification and segmentation tasks^7,44–46^. Other techniques used for uncertainty estimation in OCT images include variational inference^46,47^ and model ensembles^16,19^.

Several works have already studied the OOD detection approaches for medical images, namely for digital pathology^48^, chest X-rays^28^, brain computerized tomography (CT)^49^, abdominal CT^50^, fundus imaging^27^ and dermatology^26^. In Berger et al.^28^ they compared different methods for OOD detection. Although all the methods were capable of distinguishing natural OOD images, the same was not true in the medical domain, in an experiment in which the classifiers were trained to distinguish two pathological classes from a Chest X-ray dataset while keeping one or two other classes as OOD. Interestingly, the Mahalanobis-based approach, which performed the best on the natural images, yielded worse results than the baseline in one of the settings. Also, the benefits of ensembling and applying dropout were none or negligible. The Out-of-DIstribution detector for Neural networks (ODIN) clearly stood out as the best performing method, with the improvement coming from the input perturbation and not from the temperature scaling. This study highlighted the fact that current OOD detection methods despite performing well in computer vision tasks handling natural images were not capable enough in the medical imaging domain, demonstrating that these methods’ performances do not directly translate from one domain to the other.

In Linmans et al.^48^, they compared multiple methods for OOD detection in the domain of histopathology image analysis, and concluded that the Softmax baseline^51^ behaved considerably well, despite being slightly worse than the other tested methods. Cao et al.^27^, explored different settings and image modalities to benchmark OOD detection in medical imaging, namely the detection of OOD unrelated to the task, incorrectly acquired images, and images with unseen diseases. Different OOD detection methods were studied: Softmax maximum probability, Support vector machine (SVM) on the logits of the network, the classifier (e.g., logistic regression or k-nearest neighbors) on top of network extracted features, ODIN, Mahalanobis-based, and autoencoder reconstruction. The authors concluded that current methods performed poorly when detecting correctly acquired images that are not represented in the training data. Likewise, simpler approaches such as a classifier trained on the inlier features performed on par with more complex methods. This again highlights the conclusions from Berger et al.^28^, suggesting that current OOD methods under-perform when detecting images from the same domain that were not represented in the training data, i.e., images from unseen conditions or with artifacts. Similarly, Cao et al. concluded that OOD detection methods with promising performance on natural images^52^ performed poorly for medical images, suggesting no translation of findings between the natural and medical image domains.

Finally, Roy et al.^26^ focused on near-OOD detection of unseen conditions in dermatology. As referred by the authors, most of the literature focuses on the relatively easy task of far-OOD detection, which in their case corresponded to non-dermatology images or poor quality images, whereas the detection of previously unseen conditions, i.e., near-OOD, is a much more challenging and under-explored problem. They compared and combined different methods, namely: representation (transfer) learning, contrastive learning, ensembles, and OE with hierarchical outlier detection loss (multiple abstention classes). It was shown that OE allowed to significantly improve OOD detection, even for OOD classes not exposed during training.

**Contributions** In this study, we evaluate the efficacy and feasibility of few-shot OE to improve the robustness of a deep learning system trained for AMD staging when exposed to both near-OODs and far-OODs. To achieve this, we used a large-scale multi-center retinal OCT dataset composed of inlier cases (normal retina, iAMD, nAMD, GA) and near-OOD cases (DME, RVO, Stargardt). To evaluate far-OOD detection performance, we used eye fundus images instead of OCT. To the best of our knowledge, there have been no prior works exploring the applicability of the OOD detection methods in the context of retinal OCT classification, and in particular on the limits of the few-shot outlier exposure approaches. Our contributions are:

- A large-scale study on the applicability of deep learning for AMD classification in retinal OCT. In particular, the developed system is assessed on datasets spanning different clinical studies and acquisition settings;
- An in-depth analysis of state-of-the-art uncertainty estimation techniques for out-of-distribution (OOD) detection in retinal OCT with and without outlier exposure;
- An extensive analysis on the effect of the number of cases and their disease types on the OOD detection performance in a few-shot outlier exposure scenario.

## Materials and methods

The workflow of our study was as follows (Fig. 1). First, we fine-tuned a pre-trained, modern CNN for the classification of retinal OCT B-scans in the four AMD screening classes of interest: normal, iAMD, nAMD and GA. The CNN backbone used in all our experiments is EfficientNetV2-B0^53^, currently one of the best-performing architectures for natural image classification. EfficientNetV2-B0 was chosen given its highly competitive performance and reduced number of parameters compared with common network architectures, having shown higher accuracy and efficiency over other CNNs on ImageNet. No modifications to the architecture were made, apart from adapting the classification head to the number of classes in the study and the type of OOD detection method applied. In our preliminary analyses, we assessed a 3D approach for the same AMD classification task, using I3D (3D inflated Inception-V1 architecture, first pretrained on ImageNet and then on Kinetics dataset)^54^ and the improvement in the classification performance was not substantial. Given the marginal gains and the additional computational overload of training 3D in comparison to 2D models, we opted for the 2D setting. Thus, our network classifies 2D images corresponding to the central OCT B-scan resized to 224$\times$224 pixels.

Second, we evaluated several state-of-the-art OOD detection approaches. In particular, we studied different training/testing strategies commonly used to develop outlier-aware systems, which we refer to as *Methods* (see Fig. 1 for examples). We then used the inferred class-wise scores or the corresponding penultimate layer feature representation to extract an OOD score by using different *Metrics* (Fig. 1:Uncertainty/OOD score). These detection methods and OOD scoring metrics are detailed in the remainder of this section, and Table 1 shows which metrics were used with each of the studied OOD detection methods.

**Table 1:**
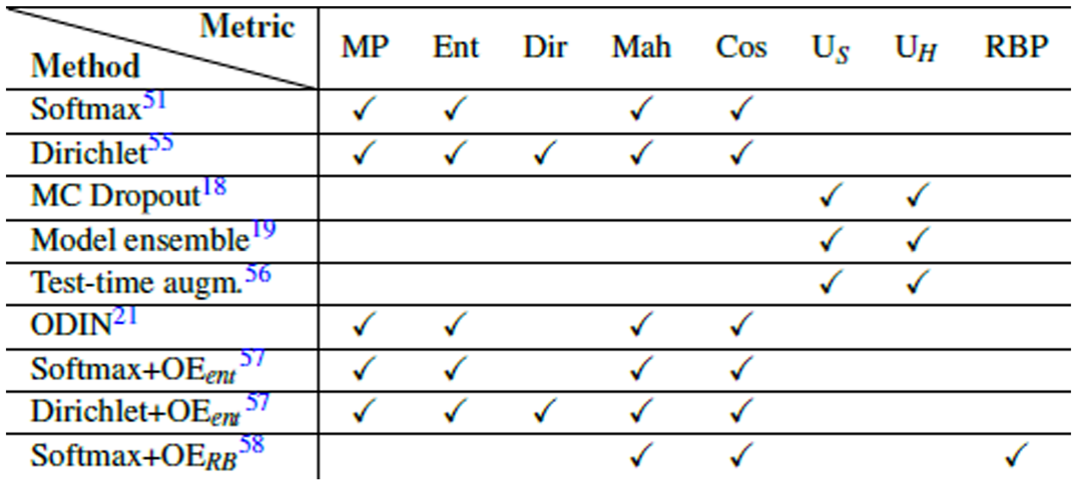
Studied methods and corresponding metrics used for out-of-distribution detection. Methods: Softmax, Dirichlet, Monte Carlo Dropout (MC Dropout), Model ensemble, Test-time augmentation (Test time augm.), Out-of-DIstribution detector for Neural networks (ODIN), Outlier exposure with entropy normalization ($\hbox {OE}_{ent}$), Outlier exposure with reject bucket ($\hbox {OE}_{RB}$). Metrics: Maximum probability (MP), Entropy (Ent), Dirichlet uncertainty (Dir), Mahalanobis distance (Mah), Cosine distance (Cos), Mean of the class variances ($\hbox {U}_S$), Mean of the class entropies ($\hbox {U}_H$), Reject bucket class probability (RBP).

### OCT dataset and study population

The OCT volumes came from baseline scans of different randomized, multicenter clinical trials available at OPTIMA Lab of MedUni Wien, and were acquired with two different manufacturers: Heidelberg Engineering ($63\%$) and Cirrus ($37\%$). Using baseline scans from clinical studies reduces the risk of having multiple pathologies on the central B-scan, facilitating both the training and the evaluation of the method. A total of 3364 OCT volumes from 2683 patients (age: 76.09±8.97, sex: 57.61% female) from the inlier classes were split patient-wise, for each class, into 70$\%$ for training, 15$\%$ for validation, and 15$\%$ for testing (detailed demographic information per class is provided in the Supplementary material, Sec. 1.2). The training set is composed of 2343 OCT images (522 normal, 783 iAMD, 726 nAMD, 312 GA), the validation set of 516 images, and the test set of 505 cases (both with the same split per class as in the training set). Regarding the near-OOD cases, a total of 295 images were included in the test set (162 DME, 19 Stargardt, and 114 RVO), and a total of 500 volumes were available for selecting the subsets for training/validation in the case of the OE techniques. Additionally, the DRIMDB^59^ dataset was used as far-OOD data test set, composed of 216 eye fundus images. All patients gave informed consent prior to inclusion in the respective multicentre clinical trials. Both the respective clinical studies as well as this analysis adhered to the tenets of the Declaration of Helsinki and the standards of Good Scientific Practice of the Medical University of Vienna. This study was approved by the Ethics Committee of the Medical University of Vienna, Vienna, Austria (EK 1246/2016).

### Out-of-distribution detection methods

**Softmax** The softmax probability is one of the most common baselines for OOD detection. It is expected that such probability value is correlated with the confidence of the class prediction, and hence it could be used for OOD detection. However, several works have shown that the softmax output tends to be overconfident, producing high probabilities even for OOD samples^19,60^. Nevertheless, Hendrycks et al.^51^ showed that despite softmax probabilities not being directly useful as confidence estimates, correctly classified samples tend to have higher maximum probabilities than erroneously classified or OOD cases.

**Evidential deep learning** The evidential deep learning approach^61^ is based on the direct modeling of the uncertainty during training. For that, the softmax output is replaced by Dirichlet distribution parameters, so that the network predictions are represented by a distribution over possible softmax outputs instead of a point estimate of that same output^55^. We thus deal with the *K* class probabilities as resulting from a Dirichlet distribution, i.e., a belief mass $b_k$ is attributed to each singleton (i.e., class label) *k*, $k\in \{1,\ldots ,K\}$, from a set of mutually exclusive singletons, and an overall uncertainty mass *u* is provided, with $u \ge 0$, $b_k \ge 0$ and $u + \sum _{k=1}^K b_k = 1$. Each $b_k$ is computed based on the evidence for that singleton $e_k$ via $b_k = {e_k}/{S}$, where *S* is the total evidence. The prediction uncertainty *u* is computed as follows:

1 $$\begin{aligned} u = \frac{K}{S} = \frac{K}{\sum _{k=1}^K(e_k + 1)}. \end{aligned}$$

The uncertainty is thus inversely proportional to the total evidence, and in the extreme case of no evidence, we have $b_k = 0, \forall k \implies u = 1$. This evidence can be modeled by a Dirichlet distribution characterized by *K* $\alpha _k$ parameters, with $\alpha _k = e_k + 1$. The probability for the class *k*, $\hat{p_k}$, is given by the mean of the Dirichlet distribution: $\hat{p_k} = \frac{\alpha _k}{S}$

**Monte Carlo (MC) dropout** MC dropout^18^ has been commonly used for approximate Bayesian inference. It consists of using dropout at test time, performing multiple predictions for a given test case, and obtaining a proxy for the true predictive posterior based on those predictions. Performing multiple predictions on the same input image can be interpreted as having an ensemble of highly similar models, and thus disagreement between the inferences is indicative of the model uncertainty^18^. This method has been widely applied^40^, mainly due to its high computational efficiency and simplicity.

**Ensembles of networks** Network ensemble is an alternative to Bayesian networks that is simple to implement, it is parallelizable and requires little hyperparameter tuning^19^. This approach consists of approximating Bayesian inference by aggregating the predictions from different networks, thus allowing to estimate model uncertainty. Given the scarcity of training data in the medical field, a common approach is to always use the entirety of the training data to train each of the models of the ensemble. Instead of introducing variability via changes to the original training data, different models are obtained by random weight initialization combined with the randomness of the data augmentation scheme.

**Test-time data augmentation** The uncertainty estimation based on test-time data augmentation consists of, at inference time, running predictions with multiple augmented versions of the original image. Similarly to the ensembles and MC Dropout, the uncertainty is estimated based on the variability of these predictions. Common data augmentation approaches include geometric and color transformations such as random crop and resize, brightness, hue, saturation, and contrast adjustments, horizontal and vertical flips, and rotation^56^.

**ODIN** The Out-of-DIstribution detector for Neural networks (ODIN)^21^ is built on the hypothesis that temperature scaling and the addition of small perturbations to the input can enhance the separation between in- and out-of-distribution samples, improving OOD detection. The softmax score after temperature scaling for the class *i* is given by $S_i(x;T) = \frac{\exp {(f_i(x)/T)}}{\sum _{j=1}^{N}\exp {(f_i(x)/T)}}$, where $\textbf{f} = (f_1,\ldots ,f_n)$ are the logits (before the softmax activation) and *T* the temperature scaling parameter, which is kept equal to 1 during training. For input processing, small perturbations are added to the input images: $\widetilde{x} = x - \varepsilon \texttt {sign}(-\nabla _x \log {S_{\hat{y}}(x;T)})$, with $\varepsilon$ corresponding to the perturbation magnitude, $\texttt {sign}$ denotes the function that returns the sign of a real number, and $\nabla _x$ stands for the gradient w.r.t. to the variable *x*. This is inspired by adversarial examples, but instead of adding perturbations to decrease the softmax score, one adds them aiming at increasing this score. The hypothesis is that this perturbation will impact more of the inliers than outliers, and thus increase their separability. OOD detection is performed by assessing the calibrated softmax score for the preprocessed image: $S_{\hat{y}}(x;T) = \max _i{S_i(\widetilde{x};T)}$, and comparing it to a threshold $\delta$, where the sample is considered an outlier if this score is $\le \delta$. The method requires a validation set with OOD samples for the choice of *T*, $\varepsilon$, and $\delta$.

**Outlier exposure (OE)** The OE approach consists of providing a limited number of OOD cases during training to improve the robustness of the model to the same (or even different^26^) types of OOD cases. In this work, we assess the two most common OE approaches: entropy normalization and reject bucket (or abstention class). For the entropy normalization approach ($OE_{ent}$), predictions on OOD images used in training are regularized against the uniform distribution to encourage high entropy posteriors on these samples^57^. In contrast, the abstention class approach^58^, or reject bucket ($OE_{RB}$), consists of adding an extra output class dedicated to the OOD cases.

### Out-of-distribution scoring metrics

**Maximum probability (MP)** This metric consists of the sample-wise maximum of the network’s probability output. Correctly classified samples tend to have higher maximum output probabilities than erroneously classified or out-of-distribution ones, making the maximum probability a reliable baseline for OOD detection.

**Mahalanobis distance** The Mahalanobis-based OOD detector^20^ is a metric that leverages the information of intermediate layer activations for OOD detection. Briefly, a class conditional Gaussian distribution is fitted to the high-dimensional features output of a given network layer using only inlier training data. At test time, the Mahalanobis distances between a test sample in the feature space and the fitted Gaussian distributions are computed, and the minimum distance is used as the OOD score. The Mahalanobis distance is defined as $\sqrt{(x-m)^T\cdot C^{-1}\cdot (x-m)}$, where *x* is the vector of the observations, *m* is the vector of mean values of the independent variables, and $C^{-1}$ is the inverse covariance matrix of the independent variables. The main intuition for dividing by the covariance is the following: if the variables are correlated, the covariance will be high and the distance will be reduced. If the variables are uncorrelated (unit variance), the Mahalanobis distance equals the Euclidean distance.

**Cosine distance** The Cosine similarity measures the similarity using the cosine of the angle between two vectors *x* and *y* in a multidimensional space. It is given by $cos(\theta ) = \frac{x\cdot y}{\left| x \right| \left| y \right| }.$ It does not measure differences in the magnitude between feature vectors, but rather the similarity in their directions. The metric used as the OOD score for a given sample is given by $1 - \max (X)$, where *X* is the vector of all the Cosine similarities computed between the test sample and the centers of each of the inlier classes, and is herein referred to as Cosine distance.

**Entropy** Shannon’s entropy is a measure of uncertainty of a discrete probability distribution and is given by: $H = -\sum _{c=1}^K \left( p(y=c) \ln (p(y=c)\right)$ where *c* is the class index, $p(y=c)$ is the network’s output probability for class *c* and *K* is the number of classes in study. The entropy $H=0$ if and only if exactly one class has a probability equal to 1 and the rest have a probability of 0. *H* is maximized when $p(y=c)~\forall c \in \{0,\ldots ,K\}$ are all equal.

**Dirichlet uncertainty** For the Dirichlet-based approach, the uncertainty taken from Eq. 1 is also considered. It is inversely proportional to the amount of total evidence collected for that image, corresponding to 1 in the extreme case of zero evidence.

**Mean of the class variances and entropies** In the Dropout, Ensemble, and Test time augmentation cases, the prediction uncertainty can be obtained from the behavior of the network across multiple predictions for the same image. A common method for prediction uncertainty is to measure how much the output probability for each class varies among the multiple inferences. For instance, $U_S$, which corresponds to the mean of the class variances for the image, is given by: $U_{S}(x) = \frac{1}{C}\sum _{c=1}^K {\sigma _c}^2$ where *x* is the image, *C* the number of classes and ${\sigma _c}^2$ are the class variances. Instead of the class variances, one can use $U_H$, the mean of the class normalized entropies, $H_c$: $U_H(x) = \frac{1}{C}\sum _{c=1}^C{H_c}$, with $H_c = \sum _{i=1}^N\hat{y}_{c,i}(x)\log _N{\hat{y}_{c,i}(x)}$, *N* being the number of networks/predictions and $\hat{y_i}$, the softmax probabilities.

**Reject bucket probability** The reject bucket probability (RBP) is the network’s softmax output probability for the reject bucket class and is used to evaluate the OOD detection performance of the $\hbox {OE}_\text {RB}$ method.

## Experiments

Let (*x*,*y*) be an input B-scan image and $y\in \{1,\ldots ,K\}$ the corresponding OCT class label. The inlier dataset $\mathscr {D}^{in}$ is composed of the central B-scans from normal, iAMD, nAMD and GA OCT volumes. The near-outlier dataset $\mathscr {D}^{out}_{near}$ is composed of the central B-scans from DME, RVO and Stargardt OCT volumes. We also used a dataset with eye fundus images as a far-outlier class $\mathscr {D}^{out}_{far}$. Depending on the OOD detection method studied, different OOD scoring metrics were considered (Table 1).

For the assessment of the performance of detecting near-OOD (all three outlier classes pooled together), since the near-OOD classes are imbalanced in the test set due to data availability, the computation of the AUC for the near-OOD was performed via class-balancing bootstrapping: 100 different subsets were randomly selected from the pool of near-OOD images, with the number of images per class equal to the one of the least represented class, and the AUC values obtained for these subsets were averaged.

### Baseline AMD detection and staging

We evaluated the performance of the state-of-the-art CNN for AMD detection and staging without considering OOD cases. The goal of this experiment is two-fold: 1) to confirm the viability of the used network architecture for this classification task, and, 2) to provide a baseline classification performance, which should not be degraded when augmenting it with OOD detection capabilities.

For this experiment, both development and test data were derived from $\mathscr {D}^{in}$. ImageNet-pretrained EfficientNetV2-B0 was fine-tuned with the categorical cross-entropy loss after softmax-activated logits with no OE.

### Near- versus far-OOD detection

We compared the OOD detection performance of the model for near (DME, RVO, Stargardt) and far (eye fundus) outliers. In particular, we aimed at identifying which uncertainty approaches are the most appropriate for OOD detection in OCT images, as well as to confirm that there is no performance degradation of the OOD detection task for near-OOD cases. For this experiment, the training and validation sets were derived from $\mathscr {D}^{in} \cup \mathscr {D}^{out}_{near}$, whereas the test set is composed of samples from either $\mathscr {D}^{in} \cup \mathscr {D}^{out}_{near}$ or $\mathscr {D}^{in} \cup \mathscr {D}^{out}_{far}$, for the near- or far-OOD detection, respectively.

### Few-shot outlier exposure OOD detection

We evaluated the performance of the OE method as a function of the number of different near-OOD samples shown per class during training. This experiment aimed at: 1) understanding the trade-off between OOD detection performance and the number of exposed outliers, 2) studying the influence of exposing one OOD class to the overall OOD detection performance and 3) qualitatively assessing changes that this few-shot OE introduces on the classification feature space. For this experiment, the training set was always a subset of $\mathscr {D}^{in} \cup \mathscr {D}^{out}_{near}$. In particular, we studied the performance of the model exposed only to DME ($\mathscr {D}^{in} \cup \mathscr {D}^{out}_{DME}$), RVO ($\mathscr {D}^{in} \cup \mathscr {D}^{out}_{RVO}$), or Stargardt ($\mathscr {D}^{in} \cup \mathscr {D}^{out}_{Stargardt }$) as well as all outlier classes together ($\mathscr {D}^{in} \cup \mathscr {D}^{out}_{near}$). The number of unique outliers exposed per class during training was $|{D}^{out}_{outlier}|= 2,\,4,\,8, \text {or}~16$. The metrics used for the computation of the AUC were the Entropy or the Cosine similarity for the $\hbox {OE}_\text {ent}$, and the RBP or the Cosine similarity for the $\hbox {OE}_\text {RB}$ method.

#### Statistical analysis

Tests were performed to evaluate the statistical difference between the different tested OOD detection methods and metrics. When necessary, bootstrapping with 100 resamplings was used for estimating the sample distribution. For the OE experiments, tests were performed comparing OE methods ($\hbox {OE}_{RB}$ and $\hbox {OE}_{ent}$), OOD detection metrics (Entropy, Cosine distance, and RBP), number of exposed volumes (0, 2, 4, 8 or 16) and exposed classes (DME, Stargardt, RVO or all). For that, the Wilcoxon Rank Sum Test was applied to the OOD detection AUCs from these models and the “p”-value resulting from the performed statistical tests is reported. The report of relevant statistical tests is provided in the Supplementary material (Sec. 1.3 and 1.4).

### Training details and hyperparameter settings

Data was augmented during training by randomly applying horizontal flipping, rotations (between $-$20 and 20 degrees), zooming (from 80$\%$ to $105\%$ of the image size), addition of Gaussian noise (standard deviation from 0 to 0.08), brightness modifications ($-$10 to 10) and contrast normalization (strength from 0.7 to 1.3). The model was trained with a batch size of 32, with class balancing, SGD optimizer, with an initial learning rate (LR) of $10^{-4}$, and LR reduction on the plateau of the validation loss (reduction by a factor 0.1 until reaching a minimum of $10^{-8}$), with a patience of 5 epochs. The maximum number of training epochs was set to 100, and training stopped when the best performance on the validation set was reached, with a patience of 15 epochs.

For the ensemble, 20 different models were trained with different weight initialization of the non-pretrained part of the CNN. For the MC dropout and Test-time augmentation, 20 inferences were performed for each trained network. For the Mahalanobis distance computation, a 100D feature space (after the penultimate fully connected layer) was used for fitting the Gaussian distributions. In the case of ODIN, for selecting the *T* and $\epsilon$ parameters, a validation set composed of inlier and near-OOD images was used. The inlier images corresponded to the validation set used during training, and 300 additional images from the near-OOD classes. The grid search was performed for $T\in [1,2,5,10,20,50,100,200,500,1000]$ and $\epsilon$ ranging from 0 to 0.1, by sampling at equal intervals 21 points from [0, 0.04] and 10 points from [0.04, 0.1].

Regarding OE, the frequency of exposure of the network to the outlier samples was set as follows: approximately 50% of the batches were exposed to outliers, and the number of outlier samples per batch ranged from 1 to N, with N=min(expected #samples for a balanced batch, #available outlier images). Experiments were performed using 1, 2, 4, 8, or 16 images from each outlier class. Five experiments were performed for each number of exposed outliers, using different sets of outlier images each time, i.e., varying the exposed outlier images, and keeping the inlier training set the same.

For Softmax and Dirichlet networks (no OE) and for Dropout, in order to have a robust estimation of the performance, 10 training runs were performed, with random weight initialization using an identical training set, and the mean of the performance metrics for the different runs was computed. For the remaining, more computationally demanding techniques, e.g. model ensembling and ODIN, a single run was performed. All the experiments were performed using Python 3.8.8, TensorFlow, and Keras 2.5.0 on a PC workstation housing NVIDIA GTX3080 GPU, Intel(R) Core(TM) i7-10700K CPU 3.80GHz, and 32 GB of RAM.

## Results and discussion

### Baseline AMD detection and staging

**Figure 2:**
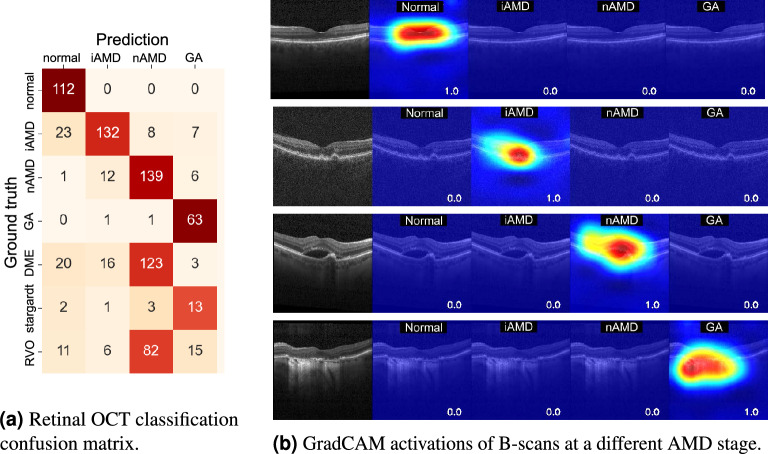
Classification confusion matrix and GradCAM for the baseline method (softmax) for OCT images showing different retinal pathologies. From top to bottom: normal, iAMD: Early/intermediate age-related macular degeneration (AMD), nAMD: Neovascular AMD, and GA: geographical atrophy.

The confusion matrix of the baseline AMD classification model, i.e. without OE or adaptations in the training scheme, is shown in Fig. 2a. The mean macro-area under the curve (AUC) for the baseline Softmax method was 0.981. The classification AUCs of the models adapted for OOD detection with different methods showed to be fairly close to each other, with a mean AUC of 0.978 across all these methods, ranging from 0.971 to 0.983. This indicates that the backbone network was capable of reliably detecting and classifying different AMD stages. Furthermore, the Gradient-weighted Class Activation Mapping (GradCAM)^62^ (Fig. 2b) showed that the model output was indeed influenced by pathological regions, confirming that the high performance was not due to, e.g., overfitting to spurious artifacts or to specific acquisition settings.

### Near- versus far-OOD detection

Table 2 shows the OOD detection performance for near-OOD, i.e., images with DME, Stargardt, and RVO disease, and Table 3 for the far-OOD cases, i.e., eye fundus images. Please refer to Table 1 for the summary of the studied uncertainty estimation methods and the corresponding metrics. The majority of the methods are unsupervised in regard to the outliers, apart from the ones involving OE and ODIN. The OE approaches used four cases per near-OOD class for exposure during training. In a later subsection, the impact of the number of samples and classes used in the exposure will be thoroughly analysed.

The best method for near-OOD detection was Softmax with ${\textrm {OE}}_{\textrm{RB}}$ with the RBP metric (AUC 0.935), followed by the same method with the Cosine metric (AUC 0.911). For the far-OOD detection, several methods obtained an almost perfect AUC$\approx 1$ when using the Cosine metric. Similarly to other studies^27,28^, it is clear that far-OOD detection is a trivial task in comparison to near-OOD detection. From the studied metrics, the Cosine distance was clearly superior for separating inliers and outliers, showing a better performance for the near-OOD detection than the remaining metrics (except RBP) and an almost perfect detection of the far-OOD cases. Indeed, except for the Mahalanobis and Cosine distances, all the metrics are based only on the output of the network, which results from a non-linear combination of the elements of the feature vector in the penultimate layer of the network. This compression seems to eliminate information relevant for OOD detection, suggesting that working in the feature domain instead of the logits domain is a better approach for this task. The notable exception is of course the RB approach, which achieves high near-OOD detection performance because in this scenario the network is directly trained for OOD detection task. Interestingly, OE, particularly in the RB setting, increases the performance of the Cosine distance for near-OOD detection without degrading the far-OOD detection performance, thus suggesting a better separation of the classes in the feature space. The same does not hold for the RBP, which, in contrast, fails for far-OOD detection. The suggested lack of generalization of the network with RBP across near and far-OODs also points to a higher robustness of the Cosine metric, and hence the superiority of utilizing the feature space of the penultimate layer for OOD detection in retinal OCT.

Given that our results indicate that CNN feature space-based metrics are better suited for OOD detection in OCT retinal images, one would expect the Mahalanobis distance to behave similarly to the Cosine distance. However, this was not the case, likely due to the assumption that the samples in the feature space follow a multivariate Gaussian distribution, which was not satisfied and consequently limited the OOD detection capability of that metric.

#### Near-OOD detection

For the near-OOD study (Table 2), the Cosine distance clearly stood out as the best metric for the separation of inliers/outliers, with the Entropy showing overall better or at least in-line performance with the Maximum Probability metric. Specifically, for the Softmax network, the Mahalanobis distance metric provides a slightly worse separation compared to the Entropy and the Maximum probability, which can be justified by the reasons stated above.

Method-wise, there is a clear superiority of the $\hbox {OE}_{RB}$ method when compared to other approaches, including other OOD-supervised methods such as $\hbox {OE}_{ent}$ and the ODIN. This is in line with the previous work, where the reject bucket was shown to be more effective than entropy normalization^57^. Nevertheless, when considering the Entropy metric, $\hbox {OE}_{ent}$ improved the near-OOD detection AUCs both for the Softmax (from 0.706 to 0.800 (p<.001) and for the Dirichlet (from 0.657 to 0.740 (*p*<.001)) networks.

Based on the obtained results, retinal OCT OOD detection should be done considering the feature space provided by the penultimate layer of the CNN. Even in cases where no OE is performed, both Softmax and Dirichlet show a very large improvement when using the Cosine metric compared to the Entropy metric performance, with the Softmax improving the AUC from 0.706 to 0.851 (*p*<.001) and the Dirichlet from 0.657 to 0.817 (*p*<.001). This puts these methods on par with the other approaches, apart from the Softmax with OE, which shows a Cosine-based AUC of 0.900 and 0.911 for $\hbox {OE}_{ent}$ and $\hbox {OE}_{RB}$, respectively.

In a scenario where the feature space is not accessible, Entropy is the most reliable method for near-OOD detection. For this metric in particular, the performance of ODIN follows OE methods, being the third-best method for near-OOD detection. This is expected because it is also a supervised approach concerning the outliers. The temperature scaling follows, which is similar to the ODIN but without the application of the perturbation on the images. Dropout, Test-time augmentation, and Ensemble behave similarly, and also in line with the baseline Softmax approach, and do not seem to justify their extra computational burden. Finally, the results suggest that the Dirichlet approach, despite its higher complexity, does not bring benefit to this task of OOD detection when compared with the simpler Softmax.

**Table 2:** Area under the ROC curve (AUC) for OOD detection of the analyzed methods based on different OOD scoring metrics for near-OOD (DME, Stargardt, RVO).

	MP	Ent	Dir	RBP	$U_H$	$U_S$	Mah	Cos	Best
Softmax	0.691	0.706					0.656	**0.851**	0.851
Dirichlet	0.658	0.657	0.647				0.635	**0.817**	0.817
MCDropout	0.697	**0.715**			0.713	0.678			0.715
Ensemble	0.701	**0.717**			0.716	0.691			0.717
TTAug	0.693	**0.714**			0.712	0.667			0.714
TempScal	0.740	0.770					0.720	**0.845**	0.845
ODIN	0.773	0.790					0.593	**0.850**	0.850
Dirichlet+$\hbox {OE}_{ent}$	0.739	0.740	0.731				0.614	**0.802**	0.802
Softmax+$\hbox {OE}_{ent}$	0.780	0.800					0.616	**0.901**	0.901
Softmax+$\hbox {OE}_{RB}$	0.719	0.811		**0.935**			0.639	0.911	0.935

Bold highlights the highest value for each of the methods.

**Table 3:** Area under the ROC curve (AUC) for OOD detection of the analyzed methods based on different OOD scoring metrics for far-OOD (eye fundus images) detection.

	MP	Ent	Dir	RBP	$U_H$	$U_S$	Mah	Cos	Best
Softmax	0.613	0.614					0.894	**0.994**	0.994
Dirichlet	0.810	0.793	0.644				0.813	**0.996**	0.996
MCDropout	0.862	0.825			0.618	**0.891**			0.891
Ensemble	0.968	**0.991**			0.210	0.980			0.991
TTAug	0.946	**0.976**			0.353	0.900			0.976
TempScal	0.380	0.374					0.896	**0.994**	0.994
ODIN	0.386	0.380					0.915	**0.997**	0.997
Dirichlet+$\hbox {OE}_{ent}$	0.805	0.790	0.660				0.849	**0.994**	0.994
Softmax+$\hbox {OE}_{ent}$	0.621	0.622					0.907	**0.994**	0.994
Softmax+$\hbox {OE}_{RB}$	0.589	0.623		0.671			0.899	**0.992**	0.992

Bold highlights the highest value for each of the methods.

### Few-shot outlier exposure OOD detection

The inlier classification performance without OE and for OE with different numbers and classes of exposed outliers was similar, with a mean AUC considering all cases of 0.980, ranging from 0.972 to 0.985. Thus, the inclusion of OE did not hinder the AMD classification performance. This is essential, as we do not want to compromise the target disease detection performance when increasing the OOD detection capability of the model.

#### Effect of the number of exposed outliers

**Figure 3:**
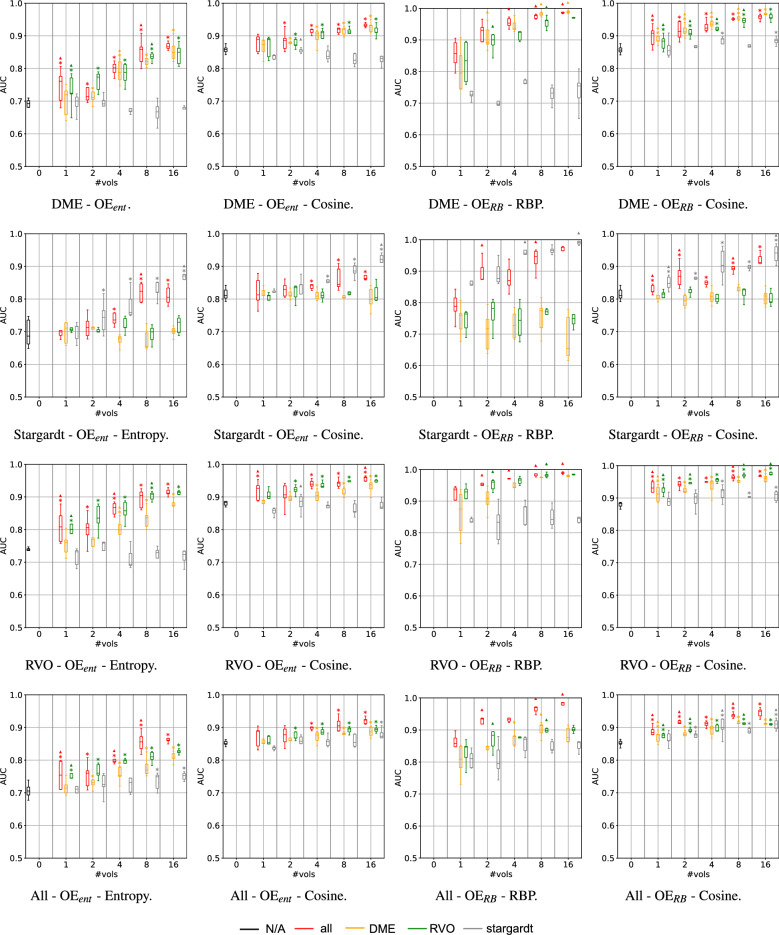
OOD detection performance for each of the near-OOD classes using entropy-based and reject bucket (RB) Outlier Exposure (OE) methods based on different metrics (Entropy, Cosine and RBP), across a growing number of exposed outlier cases ($\#vols$). The colors identify the class of the exposed outliers, with “all” denoting that $\#vols$ of each of the outlier classes were exposed during training, and “N/A” that no volumes were exposed. Each row corresponds to the detection performance of each of the outlier classes (DME, Stargardt, and RVO), and each column to a different method-metric combination. * indicates that that model is statistically significantly (*p* < .05) better than the baseline (no exposure, #vols=0); $\blacktriangle$ indicates that that model is statistically significantly (**p** < .05) better than the one trained with the immediately smaller tested number of volumes for the same exposed class.

The evolution of the OOD detection performance with the increase of the number of exposed images per near-OOD class is shown in Fig. 3. Considering the Entropy and the RBP metrics, the reject bucket-based exposure approach ($3^{rd}$ column of Fig. 3) is better in detecting near-OOD cases than the entropy normalization ($1^{st}$ column), regardless of the number of cases exposed. For instance, considering 8 exposed outliers per class (all classes exposed), $OE_{RB}$ provides an increase of 14.5$\%$, 14.1$\%$ and 9.9$\%$ in the AUCs for detecting DME, Stargardt, and RVO (*p*=.009 for all), respectively, compared to $OE_{ent}$. However, when considering the Cosine metric ($2^{nd}$ and last columns of Fig. 3), this difference is attenuated, and both methods ($\hbox {OE}_{ent}$ and $\hbox {OE}_{RB}$) show very high OOD detection performance overall, which is in line with the observed in Table 2. For the 8 exposed outliers per class, the gains of $\hbox {OE}_{RB}$ compared to $\hbox {OE}_{ent}$ are of 3.17%, 4.2% and 2.3% for DME, Stargardt, and RVO (*p*=.047, *p*=.175 and *p*=.028), respectively. Particularly for the exposure with very few OOD cases, the performance of $\hbox {OE}_{ent}$ raises significantly when using the Cosine metric instead of the Entropy for the $OE_{ent}$ (1st and 2nd columns). For only a single exposed outlier per class, the performance raises by 17.8%, 15.9%, and 13.3% (*p* = .009, *p* = .009 and *p* = .047) for the referred classes, whereas for the 8 outliers case, these gains are reduced to 8.4%, 5.1% and 5.3% (*p* = .009 for all), respectively. Overall, there is a tendency of increasing OOD detection performance as more cases are used for exposure during training. For the $OE_{RB}$, a single case of an outlier class is sufficient to significantly improve the OOD detection performance when comparing with the baseline softmax, especially with RBP metric, with improvements of 24.8%, 12.9%, and 24.3% comparing with no OE for DME, Stargardt, and RVO detection (*p* = .002, **p** = .010 and *p* = .002), respectively (considering the baseline as the network with no exposure and the Entropy score).

#### Effect of the exposed outlier classes

Regarding the type of classes used in the exposure, one can observe on the plots from Fig. 3 that exposing only the Stargardt class does not improve the DME and RVO detection performance, regardless of the number of exposed cases, and vice-versa. In fact, for the $\hbox {OE}_{ent}$, showing Stargardt cases led to DME detection AUCs that differ from $-$3.4% to 1.0% relatively to the no OE baseline, and for $\hbox {OE}_{RB}$, the difference ranges from 0.5% to 3.3%. Similarly, in the setting where DME is used in the OE, the Stargardt detection AUCs differ from $-$3.5% to 2.2% relatively to the no OE setting for the $\hbox {OE}_{ent}$, and from $-$2.5% to 1.0% for $\hbox {OE}_{RB}$. Indeed, the higher similarity between the DME and RVO classes justifies these results since nAMD (inlier), DME, and RVO (outliers) commonly present fluid in the retina as a disease manifestation. In the scenario where the network is trained solely with normal, iAMD, nAMD, and GA classes, it can be justifiable that the model simply learns to classify a volume as nAMD if fluid is present since fluid only appears in the training for nAMD cases. When the network is tested also in DME and RVO cases, it associates most of these cases with nAMD with relatively low uncertainty since it learned an association between fluid and nAMD, and both DME and RVO may also present fluid (Fig. 2a). Applying OE, which includes DME and/or RVO cases, exposing the network to a small number of examples of these similar disease classes during training, helps the network to learn that nAMD is not the only class with fluid manifestations and to better distinguish between these classes (Fig. 3). In contrast, exposure to Stargardt disease, since it does not manifest fluid, does not contribute to an improvement in outlier detection of the other two fluid-based diseases.

#### Global near-OOD detection

The balanced near-OOD detection performance (computed by bootstrapping as described in the Experiments section) across all OOD classes in our study is shown in the last row of Fig. 3. Considering the OOD score based on the Cosine similarity, with 8 outliers exposed per class, the global near-OOD detection performance for the $\hbox {OE}_{RB}$ improves by approximately 10$\%$ compared to no OE (*p*=.002), reaching an AUC of 0.937. Using the RBP metric, the OOD detection AUC for 8 exposed outliers is 0.964. The performance drops slightly if using only 4 samples per class for OE, with AUCs of 0.935 and 0.9105 using the RBP and the Cosine metrics, respectively. When using a single sample per class, these AUCs are reduced to 0.852 and 0.883, respectively. Thus, considering the RBP metric, using 8 outliers instead of a single case per class leads to an improvement of 13.2% in the OOD detection AUC (*p* = .009), whilst when using the Cosine metric the increase is of 6.1% (**p** = .009).

In summary, regardless of the exposed class, there is a trend of increasing OOD detection performance as the number of exposed cases increases. As discussed before, exposing Stargardt has the least contribution to the overall detection performance. As stated above, this effect is less noticeable when the Cosine metric is used, where all exposed classes seem to provide a more similar OOD detection performance.

#### Feature visualization and class separation

**Figure 4:**
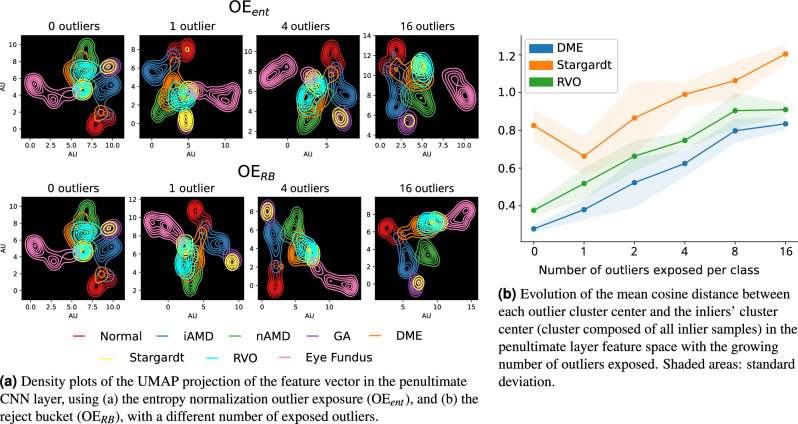
Analysis of the features from the OE networks’ penultimate layer for the different classes in the test set and the different number of exposed outliers. Normal, iAMD, nAMD and GA are the inlier classes. DME, Stargardt, and RVO are near-outliers and Eye Fundus is a far-outlier.

Figure 4 shows the penultimate layer Uniform Manifold Approximation and Projection (UMAP) feature projection^63^ on the test set $\mathscr {D}^{out}_{all}$ of a model trained using the reject bucket and the entropy normalization strategies for a different number of exposed outliers. First, note how $\mathscr {D}^{out}_{far}$ (eye fundus image) is always well separated from the inlier classes, regardless of the number of outliers and the exposure approach. Indeed, this corroborates the previous results that far-OOD detection was an easier task than near-OOD detection. Second, it is shown that the reject bucket tends to promote a better intra- and inter-class separation of the outlier classes in comparison to the entropy normalization. Finally, it is also visible that increasing the number of OOD samples per class also promotes a better separation of all classes. As expected, increasing the number of samples leads to a better representation of the pathomorphological diversity of the OOD classes, facilitating the convergence of the network to a better local optimum. In addition, in the baseline model maps, i.e., without OE, there is a large overlap between the DME and RVO classes which is expected since these classes present similar visual manifestations. The closest inlier class is the nAMD, which is in line with the expectation due to the presence of fluid, as discussed previously. Stargardt and GA clusters also show a large overlap, likely due to their similar disease manifestation.

Quantitatively, Fig. 4b shows the evolution of the Cosine distance between the classes with a different number of exposed outliers. The distance is computed between the center of the cluster composed of each of the near-OOD classes to the center of the cluster composed of all the inlier classes. As expected, one can observe the tendency of increasing the distance between OOD and inliers with the increase in the number of OOD cases exposed during training.

The above suggests that Cosine distance is a robust compromise for OOD detection of both known and unknown classes and should thus be considered as an alternative to the RBP in OE approaches, especially in the few-shot OOD exposure scenarios. The Cosine metric provides a better separation between unknown outliers (i.e. outlier classes not seen during training) and the inliers than the RBP and has a comparable performance for known outliers. As mentioned above, this should result from the higher complexity and thus representation capacity of the penultimate layer feature space.

## Conclusions

We presented an in-depth analysis of state-of-the-art uncertainty estimation techniques for OOD detection in retinal OCT imaging, with and without OE. We trained a CNN for automated AMD detection and staging, a leading cause of blindness worldwide. The datasets used for the development and testing of the approaches were diverse, composed of OCT volumes spanning multiple clinical studies and different acquisition settings. We achieved a high macro-average AUC of 0.981 for the classification of normal, iAMD, nAMD or GA using the Softmax (baseline) method.

Regarding near-OOD detection, the methods that involved supervision at the outlier level, such as ODIN and OE, outperformed the remaining ones. However, the Cosine distance metric largely improved the performance of almost all uncertainty estimation methods when compared with Maximum probability or Entropy-based metrics, reducing the gap between the unsupervised and the supervised methods. The OOD detection capability is thus particularly robust when using the Cosine distance as an OOD metric, which should result from the higher representation capacity of the last CNN extracted feature space compared to the output probabilities.

We also studied the limits of OE for retinal OCT screening. We showed that providing a very limited number of near-OOD cases (*few-shot OE*) is already sufficient to substantially increase the performance of the system for OOD detection. In general, increasing the number of outliers within the exposure set improved the OOD detection performance, with an overall near-OOD detection performance greater than 90$\%$ when using 16 volumes per class for the OE methods with the Cosine metric.

In summary, from our experiments, the Cosine distance proved to be a robust choice for OOD detection of both known and unknown disease classes and should be considered as an alternative to the Reject bucket probability in OE approaches, especially in the few-shot OE approaches. The inclusion of these methodologies will substantially improve the reliability and consequently, the trustworthiness of the resulting deep learning-based diagnostic systems in the context of retinal OCT, facilitating their translation from bench to bedside.

### Supplementary Information

Supplementary Information.

## Data Availability

The datasets used to train and evaluate our method cannot be shared at the current time due to data confidentiality agreements and privacy constraints. Please contact the author Hrvoje Bogunović for any requests.
