# Supplementary material for: Few-shot out-of-distribution detection for automated screening in retinal OCT images using deep learning
Source: Sci Rep. 2023 Sep 27;13:16231. doi: 10.1038/s41598-023-43018-9 (PMC10533534; doi:10.1038/s41598-023-43018-9)
Supplement: Supplementary file 1 — Supplementary Information. [file 41598_2023_43018_MOESM1_ESM.pdf]

1 Supplementary material

1.1 Overview of OCT classification datasets

Table 1. Characteristics of datasets used for the development of retinal OCT classification methods.

Method	2D/3D	#Classes	#Patients	#Scans (training/test or total)
Rasti *et al.* (2018) [1]	2D	3 (normal, drusen, DME)	-	7 389
Fang *et al.* (2019) [2]	2D	4 (normal, drusen, CNV, DME)	4 686	83 484
Wu *et al.* (2020) [3]	2D	4 (normal, drusen, CNV, DME)	-	136 933/6 124
Tsuji *et al.* (2020) [4]	2D	4 (normal, drusen, CNV, DME)	4 686	83 484/1 000
Kermany *et al.* (2018) [5]	2D	4 (normal, drusen, CNV, DME)	4 686	108 309/1 000
Perdomo *et al.* (2019) [6]	2D	3 (normal, AMD, DME)	-	337/122
Lee *et al.* (2017) [7]	2D	2 (normal, AMD)	9 285	80 839/20 163
Farsiu *et al.* (2014) [8,9]	3D	2 (normal, drusen)	384	384
Sun *et al.* (2020) [10]	3D	2 (AMD, DME)	-	384/910
Ours	2D	4 (normal, iAMD, nAMD, GA)	2683	2343/505

[1] Rasti, R., Rabbani, H., Mehridehnavi, A. & Hajizadeh, F. Macular OCT Classification Using a Multi-Scale Convolutional Neural Network Ensemble. *IEEE Transactions on Med. Imaging* **37**, 1024–1034, 10.1109/TMI.2017.2780115 (2018)
[2] Fang, L. *et al.* Attention to Lesion: Lesion-Aware Convolutional Neural Network for Retinal Optical Coherence Tomography Image Classification. *IEEE Transactions on Med. Imaging* **38**, 1959–1970, 10.1109/TMI.2019.2898414 (2019)
[3] Wu, J. *et al.* AttenNet: Deep Attention Based Retinal Disease Classification in OCT Images. In *MultiMedia Modeling, 26th International Conference, MMM*, vol. 281, 565–576, 10.1007/978-3-030-37734-2{_}46 (Daejeon, 2020)
[4] Tsuji, T. *et al.* Classification of optical coherence tomography images using a capsule network. *BMC Ophthalmol.* **20**, 1–9, 10.1186/s12886-020-01382-4 (2020)
[5] Kermany, D. S. *et al.* Identifying Medical Diagnoses and Treatable Diseases by Image-Based Deep Learning. *Cell* **172**, 1122–1131, 10.1016/j.cell.2018.02.010 (2018)
[6] Perdomo, O. *et al.* Classification of diabetes-related retinal diseases using a deep learning approach in optical coherence tomography. *Comput. Methods Programs Biomed.* **178**, 181–189, 10.1016/j.cmpb.2019.06.016 (2019)
[7] Lee, C. S., Baughman, D. M. & Lee, A. Y. Deep Learning Is Effective for Classifying Normal versus Age-Related Macular Degeneration OCT Images. *Ophthalmol. Retin.* **1**, 322–327, 10.1016/j.oret.2016.12.009 (2017)
[8] Farsiu, S. *et al.* Quantitative Classification of Eyes with and without Intermediate Age-related Macular Degeneration Using Optical Coherence Tomography. *Ophthalmology* **121**, 162–172, 10.1016/j.ophtha.2013.07.013 (2014)
[9] Apostolopoulos, S., Ciller, C., De Zanet, S. I., Wolf, S. & Sznitman, R. RetiNet: Automatic AMD identification in OCT volumetric data. *arXiv* 1–14 (2016)
[10] Sun, Y., Zhang, H. & Yao, X. Automatic diagnosis of macular diseases from OCT volume based on its two-dimensional feature map and convolutional neural network with attention mechanism. *J. Biomed. Opt.* **25**, 1–15, 10.1117/1.jbo.25.9.096004 (2020)

1.2 Patient demographics

Table 2. Patient demographics for each inlier class from the development dataset.

Class	#Patients	Age (mean (std))	Sex (% female)	#Scans
healthy	489	70.80 (7.54)	48.74	745
iAMD	836	76.09 (9.59)	63.28	1127
nAMD	1032	77.11 (8.61)	56.00	1040
GA	326	78.78 (7.81)	61.96	452

1.3 Statistical tests - Outlier exposure

1.3.1 Detection of All outliers

Table 3. Detection of All outliers: statistical analysis (p-value) via two-sided Wilkson ranked sum test accouting for differences in the method used to train the sytem for out-of-distribution detection. Bold indicates p-value < .05. RBP: Reject Bucket Probability; Cos: Cosine; Ent: Entropy; Vols: Number of volumes; S: OOD score; M: OOD detection method ; Starg.: stargardt; EC: Exposed class; N/A: No exposure. Ent/RBP indicates Entropy OOD score for OE_{ent} and Reject Bucket Probability OOD score for OE_{RB}.

Vols	EC	S	OE_{ent} vs OE_{RB}
0	N/A	Cos	.940
1	All	Cos	.602
1	All	Ent/RBP	**.028**
1	DME	Cos	.175
1	DME	Ent/RBP	**.016**
1	RVO	Cos	.175
1	RVO	Ent/RBP	**.016**
1	Starg.	Cos	**.047**
1	Starg.	Ent/RBP	**.009**
2	All	Cos	**.028**
2	All	Ent/RBP	**.009**
2	DME	Cos	.251
2	DME	Ent/RBP	**.009**
2	RVO	Cos	**.028**
2	RVO	Ent/RBP	**.009**
2	Starg.	Cos	.251
2	Starg.	Ent/RBP	**.028**
4	All	Cos	.251
4	All	Ent/RBP	**.009**
4	DME	Cos	.117
4	DME	Ent/RBP	**.009**
4	RVO	Cos	.175
4	RVO	Ent/RBP	**.009**
4	Starg.	Cos	**.047**
4	Starg.	Ent/RBP	**.009**
8	All	Cos	.076
8	All	Ent/RBP	**.009**
8	DME	Cos	**.028**
8	DME	Ent/RBP	**.009**
8	RVO	Cos	**.047**
8	RVO	Ent/RBP	**.009**
8	Starg.	Cos	**.009**
8	Starg.	Ent/RBP	**.009**
16	All	Cos	**.016**
16	All	Ent/RBP	**.009**
16	DME	Cos	**.028**
16	DME	Ent/RBP	**.009**
16	RVO	Cos	**.009**
16	RVO	Ent/RBP	**.009**
16	Starg.	Cos	**.028**
16	Starg.	Ent/RBP	**.009**

Table 4. Detection of All outliers: statistical analysis (p-value) via two-sided Wilkson ranked sum test accouting for differences in the metric used to score sample outlierness. Bold indicates p-value < .05. RBP: Reject Bucket Probability; Cos: Cosine; Ent: Entropy; Vols: Number of volumes; S: OOD score; M: OOD detection method ; Starg.: stargardt; EC: Exposed class; N/A: No exposure.

Vols	EC	M	Ent vs Cos	RBP vs Cos
0	N/A	OE_{ent}	**.000**	-
1	Starg.	OE_{ent}	**.009**	-
1	Starg.	OE_{RB}	-	**.016**
1	RVO	OE_{ent}	**.009**	-
1	RVO	OE_{RB}	-	.076
1	DME	OE_{ent}	**.009**	-
1	DME	OE_{RB}	-	**.016**
1	All	OE_{ent}	**.009**	-
1	All	OE_{RB}	-	.251
2	Starg.	OE_{ent}	**.009**	-
2	Starg.	OE_{RB}	-	**.047**
2	RVO	OE_{ent}	**.009**	-
2	RVO	OE_{RB}	-	.251
2	DME	OE_{ent}	**.009**	-
2	DME	OE_{RB}	-	.076
2	All	OE_{ent}	**.009**	-
2	All	OE_{RB}	-	.251
4	Starg.	OE_{ent}	**.009**	-
4	Starg.	OE_{RB}	-	**.047**
4	RVO	OE_{ent}	**.009**	-
4	RVO	OE_{RB}	-	**.047**
4	DME	OE_{ent}	**.009**	-
4	DME	OE_{RB}	-	.175
4	All	OE_{ent}	**.009**	-
4	All	OE_{RB}	-	**.009**
8	Starg.	OE_{ent}	**.009**	-
8	Starg.	OE_{RB}	-	**.009**
8	RVO	OE_{ent}	**.009**	-
8	RVO	OE_{RB}	-	**.047**
8	DME	OE_{ent}	**.009**	-
8	DME	OE_{RB}	-	.917
8	All	OE_{ent}	**.028**	-
8	All	OE_{RB}	-	**.016**
16	Starg.	OE_{ent}	**.009**	-
16	Starg.	OE_{RB}	-	**.028**
16	RVO	OE_{ent}	**.009**	-
16	RVO	OE_{RB}	-	.117
16	DME	OE_{ent}	**.009**	-
16	DME	OE_{RB}	-	.117
16	All	OE_{ent}	**.009**	-
16	All	OE_{RB}	-	**.016**

Table 5. Detection of All outliers: statistical analysis (p-value) via two-sided Wilkson ranked sum test accouting for differences in the exposed out-of-distribution class. Bold indicates p-value < .05. RBP: Reject Bucket Probability; Cos: Cosine; Ent: Entropy; Vols: Number of volumes; S: OOD score; M: OOD detection method ; Starg.: stargardt; EC: Exposed class; N/A: No exposure.

Vols	S	M	Starg. vs RVO	Starg. vs DME	Starg. vs All	RVO vs DME	RVO vs All	DME vs All
1	Ent	OE_{ent}	.076	.602	.175	.251	.602	.175
1	RBP	OE_{RB}	.251	.917	.076	.465	.251	.175
1	Cos	OE_{ent}	.117	.117	.117	.754	.347	.347
1	Cos	OE_{RB}	.602	.917	.251	.917	.175	.347
2	Ent	OE_{ent}	**.047**	.754	.251	.076	.754	.602
2	RBP	OE_{RB}	**.047**	.251	**.028**	.117	.117	.076
2	Cos	OE_{ent}	.175	.602	.251	.251	.917	.465
2	Cos	OE_{RB}	**.028**	.754	**.028**	.076	.117	**.047**
4	Ent	OE_{ent}	**.028**	**.047**	**.009**	.076	.754	**.009**
4	RBP	OE_{RB}	.175	.175	**.009**	.917	**.009**	**.009**
4	Cos	OE_{ent}	.076	.251	**.009**	.251	**.047**	**.009**
4	Cos	OE_{RB}	.465	.602	.754	.754	.076	.076
8	Ent	OE_{ent}	**.009**	**.047**	**.009**	.117	**.028**	**.016**
8	RBP	OE_{RB}	**.009**	**.016**	**.009**	.602	**.009**	**.009**
8	Cos	OE_{ent}	**.028**	.076	**.009**	.117	.175	**.009**
8	Cos	OE_{RB}	**.028**	.117	**.009**	.917	**.009**	**.009**
16	Ent	OE_{ent}	**.009**	**.009**	**.009**	.076	**.009**	**.009**
16	RBP	OE_{RB}	**.016**	.076	**.009**	.347	**.009**	**.009**
16	Cos	OE_{ent}	.117	.465	**.009**	.465	**.009**	**.009**
16	Cos	OE_{RB}	.917	.917	**.016**	.602	**.009**	**.009**

Table 6. Detection of All outliers: statistical analysis (p-value) via two-sided Wilkson ranked sum test accouting for differences in the number of exposed volumes. Bold indicates p-value < .05. RBP: Reject Bucket Probability; Cos: Cosine; Ent: Entropy; Vols: Number of volumes; S: OOD score; M: OOD detection method ; Starg.: stargardt; EC: Exposed class; N/A: No exposure. * indicates cases where comparison was performed with training with 0 volumes using Entropy OOD score.

EC	S	M	0 vs 1	0 vs 2	0 vs 4	0 vs 8	0 vs 16	1 vs 2	1 vs 4	1 vs 8	1 vs 16	2 vs 4	2 vs 8	2 vs 16	4 vs 8	4 vs 16	8 vs 16
Starg.	Ent	OE_{ent}	.806	.142	.178	.050	**.003**	.175	.251	.175	**.009**	.754	.754	.175	.251	.076	.465
Starg.	RBP	OE_{RB}	**.002***	**.002***	**.002***	**.002***	**.002***	.754	**.047**	**.047**	**.047**	.251	.251	.117	.602	.917	.602
Starg.	Cos	OE_{ent}	**.007**	.327	.540	.624	.066	.117	**.016**	**.047**	.076	.917	.754	.251	.754	.251	.465
Starg.	Cos	OE_{RB}	.178	**.027**	**.007**	**.002**	**.002**	.602	.076	**.047**	**.016**	.076	**.047**	**.016**	.347	.754	.076
RVO	Ent	OE_{ent}	.050	**.003**	**.002**	**.002**	**.002**	.465	.117	**.016**	**.009**	.117	**.028**	**.009**	.117	**.009**	.117
RVO	RBP	OE_{RB}	**.002***	**.002***	**.002***	**.002***	**.002***	.076	**.016**	**.009**	**.009**	.917	.076	**.028**	**.047**	**.047**	.602
RVO	Cos	OE_{ent}	.462	**.005**	**.027**	**.002**	**.002**	.175	.076	**.009**	**.009**	.465	**.047**	**.028**	.347	.347	.917
RVO	Cos	OE_{RB}	**.037**	**.002**	**.002**	**.002**	**.002**	**.009**	**.009**	**.009**	**.009**	.465	**.028**	**.009**	**.047**	.117	.754
DME	Ent	OE_{ent}	.391	**.037**	**.005**	**.002**	**.002**	.251	.076	**.016**	**.009**	.175	**.028**	**.009**	.251	**.009**	.117
DME	RBP	OE_{RB}	**.003***	**.002***	**.002***	**.002***	**.002***	.347	**.016**	**.009**	**.009**	**.028**	**.016**	**.028**	.076	.465	.175
DME	Cos	OE_{ent}	.462	.178	.066	**.002**	**.002**	.465	.175	**.016**	**.009**	.347	.117	**.047**	.465	.251	.465
DME	Cos	OE_{RB}	.050	**.037**	**.002**	**.002**	**.002**	.754	.076	**.016**	**.009**	.175	**.028**	**.009**	.117	.175	.754
All	Ent	OE_{ent}	**.037**	**.020**	**.002**	**.002**	**.002**	.917	.117	**.009**	**.009**	.076	**.009**	**.009**	**.016**	**.009**	.602
All	RBP	OE_{RB}	**.002***	**.002***	**.002***	**.002***	**.002***	.076	**.009**	**.009**	**.009**	.465	**.009**	**.009**	**.016**	**.009**	**.047**
All	Cos	OE_{ent}	.391	.142	**.002**	**.002**	**.002**	.754	.117	.117	**.009**	.175	.117	**.009**	.754	**.028**	.251
All	Cos	OE_{RB}	**.027**	**.002**	**.002**	**.002**	**.002**	.076	**.047**	**.009**	**.009**	.465	**.009**	**.009**	**.009**	**.009**	.347

1.3.2 Detection of DME

Table 7. Detection of DME: statistical analysis (*p*-value) via two-sided Wilkson ranked sum test accouting for differences in the method used to train the sytem for out-of-distribution detection. Bold indicates *p*-value < .05. RBP: Reject Bucket Probability; Cos: Cosine; Ent: Entropy; Vols: Number of volumes; S: OOD score; M: OOD detection method ; Starg.: stargardt; EC: Exposed class; N/A: No exposure. Ent/RBP indicates Entropy OOD score for OE_{ent} and Reject Bucket Probability OOD score for OE_{RB}.

Vols	EC	S	OE_{ent} vs OE_{RB}
0	N/A	Cos	.821
1	All	Cos	.175
1	All	Ent/RBP	**.016**
1	DME	Cos	.251
1	DME	Ent/RBP	**.028**
1	RVO	Cos	.754
1	RVO	Ent/RBP	.076
1	Starg.	Cos	.076
1	Starg.	Ent/RBP	**.028**
2	All	Cos	.117
2	All	Ent/RBP	**.016**
2	DME	Cos	**.016**
2	DME	Ent/RBP	**.009**
2	RVO	Cos	**.016**
2	RVO	Ent/RBP	**.009**
2	Starg.	Cos	.117
2	Starg.	Ent/RBP	.602
4	All	Cos	.175
4	All	Ent/RBP	**.009**
4	DME	Cos	**.009**
4	DME	Ent/RBP	**.009**
4	RVO	Cos	**.047**
4	RVO	Ent/RBP	**.009**
4	Starg.	Cos	**.028**
4	Starg.	Ent/RBP	**.009**
8	All	Cos	**.047**
8	All	Ent/RBP	**.009**
8	DME	Cos	**.009**
8	DME	Ent/RBP	**.009**
8	RVO	Cos	**.016**
8	RVO	Ent/RBP	**.009**
8	Starg.	Cos	**.009**
8	Starg.	Ent/RBP	**.016**
16	All	Cos	**.009**
16	All	Ent/RBP	**.009**
16	DME	Cos	**.016**
16	DME	Ent/RBP	**.009**
16	RVO	Cos	**.009**
16	RVO	Ent/RBP	**.009**
16	Starg.	Cos	**.016**
16	Starg.	Ent/RBP	.076

Table 8. Detection of DME: statistical analysis (*p*-value) via two-sided Wilkson ranked sum test accouting for differences in the metric used to score sample outlierness. Bold indicates *p*-value < .05. RBP: Reject Bucket Probability; Cos: Cosine; Ent: Entropy; Vols: Number of volumes; S: OOD score; M: OOD detection method ; Starg.: stargardt; EC: Exposed class; N/A: No exposure.

Vols	EC	M	Ent vs Cos	RBP vs Cos
0	N/A	OE_{ent}	**.000**	-
1	Starg.	OE_{ent}	**.009**	-
1	Starg.	OE_{RB}	-	**.009**
1	RVO	OE_{ent}	**.009**	-
1	RVO	OE_{RB}	-	.347
1	DME	OE_{ent}	**.009**	-
1	DME	OE_{RB}	-	.076
1	All	OE_{ent}	**.009**	-
1	All	OE_{RB}	-	.117
2	Starg.	OE_{ent}	**.009**	-
2	Starg.	OE_{RB}	-	**.016**
2	RVO	OE_{ent}	**.009**	-
2	RVO	OE_{RB}	-	.251
2	DME	OE_{ent}	**.009**	-
2	DME	OE_{RB}	-	.465
2	All	OE_{ent}	**.009**	-
2	All	OE_{RB}	-	.917
4	Starg.	OE_{ent}	**.009**	-
4	Starg.	OE_{RB}	-	**.009**
4	RVO	OE_{ent}	**.009**	-
4	RVO	OE_{RB}	-	.754
4	DME	OE_{ent}	**.009**	-
4	DME	OE_{RB}	-	.465
4	All	OE_{ent}	**.009**	-
4	All	OE_{RB}	-	.076
8	Starg.	OE_{ent}	**.009**	-
8	Starg.	OE_{RB}	-	**.009**
8	RVO	OE_{ent}	**.009**	-
8	RVO	OE_{RB}	-	.251
8	DME	OE_{ent}	**.009**	-
8	DME	OE_{RB}	-	**.009**
8	All	OE_{ent}	**.016**	-
8	All	OE_{RB}	-	**.009**
16	Starg.	OE_{ent}	**.009**	-
16	Starg.	OE_{RB}	-	**.009**
16	RVO	OE_{ent}	**.009**	-
16	RVO	OE_{RB}	-	.175
16	DME	OE_{ent}	**.009**	-
16	DME	OE_{RB}	-	**.009**
16	All	OE_{ent}	**.009**	-
16	All	OE_{RB}	-	**.009**

Table 9. Detection of DME: statistical analysis (p-value) via two-sided Wilkson ranked sum test accouting for differences in the exposed out-of-distribution class. Bold indicates p-value < .05. RBP: Reject Bucket Probability; Cos: Cosine; Ent: Entropy; Vols: Number of volumes; S: OOD score; M: OOD detection method ; Starg.: stargardt; EC: Exposed class; N/A: No exposure.

Vols	S	M	Starg. vs RVO	Starg. vs DME	Starg. vs All	RVO vs DME	RVO vs All	DME vs All
1	Ent	OE_{ent}	.117	.602	.175	.347	.754	.175
1	RBP	OE_{RB}	**.028**	**.047**	**.016**	.602	.602	.347
1	Cos	OE_{ent}	.347	.117	**.009**	.347	.347	.347
1	Cos	OE_{RB}	.347	.117	.076	.465	.347	.754
2	Ent	OE_{ent}	**.016**	.175	.076	.175	.251	.754
2	RBP	OE_{RB}	**.009**	**.009**	**.016**	.602	.347	.602
2	Cos	OE_{ent}	**.016**	.117	.076	.754	.917	.602
2	Cos	OE_{RB}	**.009**	**.009**	**.016**	.347	.754	.754
4	Ent	OE_{ent}	**.009**	**.009**	**.009**	.602	.465	.602
4	RBP	OE_{RB}	**.009**	**.009**	**.009**	.076	.076	.465
4	Cos	OE_{ent}	**.028**	**.016**	**.009**	.754	.347	.117
4	Cos	OE_{RB}	**.009**	**.009**	**.009**	.251	.917	.347
8	Ent	OE_{ent}	**.009**	**.009**	**.009**	.347	.465	.602
8	RBP	OE_{RB}	**.009**	**.009**	**.009**	**.016**	**.047**	.117
8	Cos	OE_{ent}	**.009**	**.009**	**.009**	.754	.465	.602
8	Cos	OE_{RB}	**.009**	**.009**	**.009**	.095	.251	.251
16	Ent	OE_{ent}	**.009**	**.009**	**.009**	.602	.175	.347
16	RBP	OE_{RB}	**.009**	**.009**	**.009**	**.009**	**.009**	.754
16	Cos	OE_{ent}	**.009**	**.009**	**.009**	.347	.076	.602
16	Cos	OE_{RB}	**.009**	**.009**	**.009**	.347	.917	.175

Table 10. Detection of DME: statistical analysis (p-value) via two-sided Wilkson ranked sum test accouting for differences in the number of exposed volumes. Bold indicates p-value < .05. RBP: Reject Bucket Probability; Cos: Cosine; Ent: Entropy; Vols: Number of volumes; S: OOD score; M: OOD detection method ; Starg.: stargardt; EC: Exposed class; N/A: No exposure. * indicates cases where comparison was performed with training with 0 volumes using Entropy OOD score.

EC	S	M	0 vs 1	0 vs 2	0 vs 4	0 vs 8	0 vs 16	1 vs 2	1 vs 4	1 vs 8	1 vs 16	2 vs 4	2 vs 8	2 vs 16	4 vs 8	4 vs 16	8 vs 16
Starg.	Ent	OE_{ent}	.462	.624	.391	.270	.142	.917	.347	.175	.117	.347	.175	.117	.465	.754	.754
Starg.	RBP	OE_{RB}	**.005***	.462*	**.002***	**.020***	.050*	.175	.347	.917	.917	.117	.347	.347	**.047**	.465	.465
Starg.	Cos	OE_{ent}	**.005**	.806	.111	**.010**	.066	**.009**	.754	.602	.465	.251	**.016**	.117	.251	.754	.917
Starg.	Cos	OE_{RB}	.903	.270	**.037**	.111	**.005**	.917	.251	.465	.175	.117	.602	**.047**	.251	.917	.175
RVO	Ent	OE_{ent}	.050	**.002**	**.002**	**.002**	**.002**	.347	.076	**.009**	**.009**	.251	**.009**	**.009**	**.009**	**.028**	.465
RVO	RBP	OE_{RB}	**.002***	**.002***	**.002***	**.002***	**.002***	.076	**.016**	**.009**	**.009**	.175	**.009**	**.009**	.076	.076	.251
RVO	Cos	OE_{ent}	.540	**.020**	**.037**	**.002**	**.002**	.917	.076	**.047**	**.028**	.175	**.028**	**.016**	.602	.347	.754
RVO	Cos	OE_{RB}	.050	**.002**	**.002**	**.002**	**.002**	.076	**.009**	**.009**	**.009**	.076	**.009**	**.009**	.076	**.016**	.117
DME	Ent	OE_{ent}	.391	**.037**	**.002**	**.002**	**.002**	.754	**.016**	**.009**	**.009**	**.047**	**.009**	**.009**	.117	**.016**	.251
DME	RBP	OE_{RB}	**.002***	**.002***	**.002***	**.002***	**.002***	**.047**	**.009**	**.009**	**.009**	.117	**.009**	**.009**	**.016**	**.009**	.076
DME	Cos	OE_{ent}	.327	.086	**.020**	**.002**	**.002**	.602	**.047**	**.009**	**.009**	.117	**.016**	**.009**	.175	**.047**	.175
DME	Cos	OE_{RB}	**.005**	**.002**	**.002**	**.002**	**.002**	.076	**.016**	**.009**	**.009**	.076	**.009**	**.009**	**.009**	**.009**	.251
All	Ent	OE_{ent}	.066	**.014**	**.002**	**.002**	**.002**	.917	.076	**.016**	**.009**	.117	**.028**	**.009**	.076	**.009**	.347
All	RBP	OE_{RB}	**.002***	**.002***	**.002***	**.002***	**.002***	.175	**.009**	**.009**	**.009**	.076	**.016**	**.009**	**.047**	**.009**	**.009**
All	Cos	OE_{ent}	.221	.066	**.002**	**.002**	**.002**	.917	**.016**	**.016**	**.009**	.117	.076	**.016**	.465	**.009**	.117
All	Cos	OE_{RB}	**.020**	**.002**	**.002**	**.002**	**.002**	.347	.117	**.009**	**.009**	.465	**.009**	**.028**	.117	**.028**	.251

1.3.3 Detection of Stargardt

Table 11. Detection of Stargardt disease: statistical analysis (p-value) via two-sided Wilkson ranked sum test accouting for differences in the method used to train the sytem for out-of-distribution detection. Bold indicates p-value < .05. RBP: Reject Bucket Probability; Cos: Cosine; Ent: Entropy; Vols: Number of volumes; S: OOD score; M: OOD detection method ; Starg.: stargardt; EC: Exposed class; N/A: No exposure. Ent/RBP indicates Entropy OOD score for OE_{ent} and Reject Bucket Probability OOD score for OE_{RB}.

Vols	EC	S	OE_{ent} vs OE_{RB}
0	N/A	Cos	.940
1	All	Cos	.754
1	All	Ent/RBP	**.028**
1	DME	Cos	.917
1	DME	Ent/RBP	.117
1	RVO	Cos	.465
1	RVO	Ent/RBP	.175
1	Starg.	Cos	**.028**
1	Starg.	Ent/RBP	**.009**
2	All	Cos	**.047**
2	All	Ent/RBP	**.009**
2	DME	Cos	.117
2	DME	Ent/RBP	.754
2	RVO	Cos	.602
2	RVO	Ent/RBP	.076
2	Starg.	Cos	.117
2	Starg.	Ent/RBP	**.009**
4	All	Cos	.175
4	All	Ent/RBP	**.009**
4	DME	Cos	.602
4	DME	Ent/RBP	**.047**
4	RVO	Cos	.602
4	RVO	Ent/RBP	.602
4	Starg.	Cos	.175
4	Starg.	Ent/RBP	**.009**
8	All	Cos	.175
8	All	Ent/RBP	**.009**
8	DME	Cos	.175
8	DME	Ent/RBP	**.047**
8	RVO	Cos	.917
8	RVO	Ent/RBP	**.028**
8	Starg.	Cos	.251
8	Starg.	Ent/RBP	**.009**
16	All	Cos	.117
16	All	Ent/RBP	**.009**
16	DME	Cos	.917
16	DME	Ent/RBP	.602
16	RVO	Cos	.175
16	RVO	Ent/RBP	.175
16	Starg.	Cos	.347
16	Starg.	Ent/RBP	**.009**

Table 12. Detection of Stargardt disease: statistical analysis (p-value) via two-sided Wilkson ranked sum test accouting for differences in the metric used to score sample outlierness. Bold indicates p-value < .05. RBP: Reject Bucket Probability; Cos: Cosine; Ent: Entropy; Vols: Number of volumes; S: OOD score; M: OOD detection method ; Starg.: stargardt; EC: Exposed class; N/A: No exposure.

Vols	EC	M	Ent vs Cos	RBP vs Cos
0	N/A	OE_{ent}	**.000**	-
1	Starg.	OE_{ent}	**.009**	-
1	Starg.	OE_{RB}	-	.602
1	RVO	OE_{ent}	**.009**	-
1	RVO	OE_{RB}	-	**.009**
1	DME	OE_{ent}	**.009**	-
1	DME	OE_{RB}	-	**.028**
1	All	OE_{ent}	**.016**	-
1	All	OE_{RB}	-	.117
2	Starg.	OE_{ent}	**.047**	-
2	Starg.	OE_{RB}	-	.175
2	RVO	OE_{ent}	**.009**	-
2	RVO	OE_{RB}	-	**.016**
2	DME	OE_{ent}	**.009**	-
2	DME	OE_{RB}	-	**.028**
2	All	OE_{ent}	**.009**	-
2	All	OE_{RB}	-	.602
4	Starg.	OE_{ent}	**.028**	-
4	Starg.	OE_{RB}	-	.175
4	RVO	OE_{ent}	**.009**	-
4	RVO	OE_{RB}	-	**.047**
4	DME	OE_{ent}	**.009**	-
4	DME	OE_{RB}	-	**.016**
4	All	OE_{ent}	**.009**	-
4	All	OE_{RB}	-	.251
8	Starg.	OE_{ent}	**.009**	-
8	Starg.	OE_{RB}	-	**.009**
8	RVO	OE_{ent}	**.009**	-
8	RVO	OE_{RB}	-	**.009**
8	DME	OE_{ent}	**.009**	-
8	DME	OE_{RB}	-	**.016**
8	All	OE_{ent}	.175	-
8	All	OE_{RB}	-	.117
16	Starg.	OE_{ent}	.076	-
16	Starg.	OE_{RB}	-	**.009**
16	RVO	OE_{ent}	**.009**	-
16	RVO	OE_{RB}	-	**.047**
16	DME	OE_{ent}	**.009**	-
16	DME	OE_{RB}	-	**.016**
16	All	OE_{ent}	**.009**	-
16	All	OE_{RB}	-	.076

Table 13. Detection of Stargardt disease: statistical analysis (p-value) via two-sided Wilkson ranked sum test accouting for differences in the exposed out-of-distribution class. Bold indicates p-value < .05. RBP: Reject Bucket Probability; Cos: Cosine; Ent: Entropy; Vols: Number of volumes; S: OOD score; M: OOD detection method ; Starg.: stargardt; EC: Exposed class; N/A: No exposure.

Vols	S	M	Starg. vs RVO	Starg. vs DME	Starg. vs All	RVO vs DME	RVO vs All	DME vs All
1	Ent	OE_{ent}	.917	.754	.917	.917	.347	.917
1	RBP	OE_{RB}	**.009**	**.016**	**.047**	.754	**.047**	.117
1	Cos	OE_{ent}	**.047**	.754	.754	.347	.465	.754
1	Cos	OE_{RB}	**.016**	**.028**	.175	.754	.117	**.047**
2	Ent	OE_{ent}	.175	.465	.465	.175	.465	.917
2	RBP	OE_{RB}	**.009**	**.009**	.917	.175	**.028**	**.016**
2	Cos	OE_{ent}	.347	.251	.602	.465	.602	.347
2	Cos	OE_{RB}	**.009**	**.009**	.602	**.028**	**.016**	**.009**
4	Ent	OE_{ent}	**.009**	**.009**	.076	**.016**	.175	**.009**
4	RBP	OE_{RB}	**.009**	**.009**	**.047**	.754	**.009**	**.009**
4	Cos	OE_{ent}	**.028**	**.028**	.347	.917	**.016**	**.028**
4	Cos	OE_{RB}	**.016**	**.009**	.117	.917	.076	**.016**
8	Ent	OE_{ent}	**.009**	**.009**	.917	.347	**.009**	**.009**
8	RBP	OE_{RB}	**.009**	**.009**	.117	.754	**.009**	**.009**
8	Cos	OE_{ent}	**.009**	**.009**	.251	.076	**.028**	**.016**
8	Cos	OE_{RB}	**.009**	**.009**	.602	.602	**.009**	**.009**
16	Ent	OE_{ent}	**.009**	**.009**	**.028**	.251	**.009**	**.009**
16	RBP	OE_{RB}	**.009**	**.009**	**.009**	.251	**.009**	**.009**
16	Cos	OE_{ent}	**.016**	**.009**	.076	.465	**.028**	**.009**
16	Cos	OE_{RB}	**.009**	**.009**	.251	.917	**.009**	**.009**

Table 14. Detection of Stargardt disease: statistical analysis (p-value) via two-sided Wilkson ranked sum test accouting for differences in the number of exposed volumes. Bold indicates p-value < .05. RBP: Reject Bucket Probability; Cos: Cosine; Ent: Entropy; Vols: Number of volumes; S: OOD score; M: OOD detection method ; Starg.: stargardt; EC: Exposed class; N/A: No exposure. * indicates cases where comparison was performed with training with 0 volumes using Entropy OOD score.

EC	S	M	0 vs 1	0 vs 2	0 vs 4	0 vs 8	0 vs 16	1 vs 2	1 vs 4	1 vs 8	1 vs 16	2 vs 4	2 vs 8	2 vs 16	4 vs 8	4 vs 16	8 vs 16
Starg.	Ent	OE_{ent}	1.000	.086	**.002**	**.002**	**.002**	.175	**.009**	**.009**	**.009**	.251	**.016**	**.016**	.117	**.016**	.076
Starg.	RBP	OE_{RB}	**.002***	**.002***	**.002***	**.002***	**.002***	.175	**.028**	**.009**	**.009**	.076	**.009**	**.009**	.602	**.028**	**.016**
Starg.	Cos	OE_{ent}	.540	.221	**.014**	**.002**	**.002**	.465	**.047**	**.009**	**.009**	.175	**.016**	**.016**	.175	**.047**	.076
Starg.	Cos	OE_{RB}	**.014**	**.002**	**.002**	**.002**	**.002**	.465	**.047**	**.009**	**.009**	.117	**.009**	**.009**	.754	.347	.076
RVO	Ent	OE_{ent}	.806	.806	.270	.713	.178	.347	.251	.347	.602	.175	.917	.347	.117	.917	.175
RVO	RBP	OE_{RB}	.050*	**.027***	.111*	**.014***	**.037***	.347	.917	.347	.917	.347	.602	.754	.602	.602	.465
RVO	Cos	OE_{ent}	.221	.540	.624	.462	.903	.251	.465	**.047**	.602	.347	.917	.754	.251	.754	.465
RVO	Cos	OE_{RB}	.806	.221	.358	.540	.178	.175	.602	.347	.754	.175	.754	.117	.602	.602	.465
DME	Ent	OE_{ent}	.903	.624	.270	.221	1.000	.602	.347	.117	.754	**.016**	.117	.347	.917	**.047**	.251
DME	RBP	OE_{RB}	.066*	.806*	.178*	.066*	.713*	.347	.754	.465	.175	.602	.251	.602	.602	.251	.117
DME	Cos	OE_{ent}	1.000	.903	.540	.540	.391	.917	.602	.403	.754	.754	.465	.602	.754	.602	.754
DME	Cos	OE_{RB}	.713	.086	.540	.327	.462	.251	.917	.251	.754	.465	**.047**	.465	.251	.754	.251
All	Ent	OE_{ent}	.624	.462	.066	**.002**	**.002**	.754	.117	**.009**	**.009**	.251	**.009**	**.009**	**.009**	**.009**	.602
All	RBP	OE_{RB}	**.010***	**.002***	**.002***	**.002***	**.002***	**.028**	**.016**	**.009**	**.009**	.602	.076	**.028**	**.047**	**.028**	.117
All	Cos	OE_{ent}	.903	.221	**.014**	**.010**	**.002**	.754	.347	.175	.076	.251	.175	**.028**	.465	.076	.465
All	Cos	OE_{RB}	.086	**.005**	**.003**	**.002**	**.002**	**.047**	**.047**	**.009**	**.009**	.754	.117	.117	.076	**.028**	.347

1.3.4 Detection of RVO

Table 15. Detection of RVO: statistical analysis (p-value) via two-sided Wilkson ranked sum test accouting for differences in the method used to train the sytem for out-of-distribution detection. Bold indicates p-value < .05. RBP: Reject Bucket Probability; Cos: Cosine; Ent: Entropy; Vols: Number of volumes; S: OOD score; M: OOD detection method ; Starg.: stargardt; EC: Exposed class; N/A: No exposure. Ent/RBP indicates Entropy OOD score for OE_{ent} and Reject Bucket Probability OOD score for OE_{RB}.

Vols	EC	S	OE_{ent} vs OE_{RB}
0	N/A	Cos	.821
1	All	Cos	.754
1	All	Ent/RBP	**.028**
1	DME	Cos	.117
1	DME	Ent/RBP	**.028**
1	RVO	Cos	.117
1	RVO	Ent/RBP	**.016**
1	Starg.	Cos	.076
1	Starg.	Ent/RBP	**.009**
2	All	Cos	**.047**
2	All	Ent/RBP	**.009**
2	DME	Cos	.076
2	DME	Ent/RBP	**.009**
2	RVO	Cos	**.016**
2	RVO	Ent/RBP	**.009**
2	Starg.	Cos	.347
2	Starg.	Ent/RBP	**.009**
4	All	Cos	.347
4	All	Ent/RBP	**.009**
4	DME	Cos	**.047**
4	DME	Ent/RBP	**.009**
4	RVO	Cos	**.016**
4	RVO	Ent/RBP	**.009**
4	Starg.	Cos	**.016**
4	Starg.	Ent/RBP	**.009**
8	All	Cos	**.028**
8	All	Ent/RBP	**.009**
8	DME	Cos	**.028**
8	DME	Ent/RBP	**.009**
8	RVO	Cos	**.009**
8	RVO	Ent/RBP	**.009**
8	Starg.	Cos	**.016**
8	Starg.	Ent/RBP	**.009**
16	All	Cos	**.028**
16	All	Ent/RBP	**.009**
16	DME	Cos	**.028**
16	DME	Ent/RBP	**.009**
16	RVO	Cos	**.009**
16	RVO	Ent/RBP	**.009**
16	Starg.	Cos	**.028**
16	Starg.	Ent/RBP	**.009**

Table 16. Detection of RVO: statistical analysis (p-value) via two-sided Wilkson ranked sum test accouting for differences in the metric used to score sample outlierness. Bold indicates p-value < .05. RBP: Reject Bucket Probability; Cos: Cosine; Ent: Entropy; Vols: Number of volumes; S: OOD score; M: OOD detection method ; Starg.: stargardt; EC: Exposed class; N/A: No exposure.

Vols	EC	M	Ent vs Cos	RBP vs Cos
0	N/A	OE_{ent}	**.000**	-
1	Starg.	OE_{ent}	**.009**	-
1	Starg.	OE_{RB}	-	**.016**
1	RVO	OE_{ent}	**.047**	-
1	RVO	OE_{RB}	-	.917
1	DME	OE_{ent}	**.009**	-
1	DME	OE_{RB}	-	.251
1	All	OE_{ent}	**.009**	-
1	All	OE_{RB}	-	.602
2	Starg.	OE_{ent}	**.009**	-
2	Starg.	OE_{RB}	-	.076
2	RVO	OE_{ent}	**.009**	-
2	RVO	OE_{RB}	-	.465
2	DME	OE_{ent}	**.009**	-
2	DME	OE_{RB}	-	.754
2	All	OE_{ent}	**.016**	-
2	All	OE_{RB}	-	.251
4	Starg.	OE_{ent}	**.009**	-
4	Starg.	OE_{RB}	-	.076
4	RVO	OE_{ent}	**.009**	-
4	RVO	OE_{RB}	-	.251
4	DME	OE_{ent}	**.009**	-
4	DME	OE_{RB}	-	.754
4	All	OE_{ent}	**.009**	-
4	All	OE_{RB}	-	**.009**
8	Starg.	OE_{ent}	**.009**	-
8	Starg.	OE_{RB}	-	**.016**
8	RVO	OE_{ent}	**.009**	-
8	RVO	OE_{RB}	-	**.016**
8	DME	OE_{ent}	**.009**	-
8	DME	OE_{RB}	-	**.047**
8	All	OE_{ent}	**.009**	-
8	All	OE_{RB}	-	**.009**
16	Starg.	OE_{ent}	**.009**	-
16	Starg.	OE_{RB}	-	**.016**
16	RVO	OE_{ent}	**.009**	-
16	RVO	OE_{RB}	-	**.009**
16	DME	OE_{ent}	**.009**	-
16	DME	OE_{RB}	-	**.016**
16	All	OE_{ent}	**.009**	-
16	All	OE_{RB}	-	**.009**

Table 17. Detection of RVO: statistical analysis (p-value) via two-sided Wilkson ranked sum test accouting for differences in the exposed out-of-distribution class. Bold indicates p-value < .05. RBP: Reject Bucket Probability; Cos: Cosine; Ent: Entropy; Vols: Number of volumes; S: OOD score; M: OOD detection method ; Starg.: stargardt; EC: Exposed class; N/A: No exposure.

Vols	S	M	Starg. vs RVO	Starg. vs DME	Starg. vs All	RVO vs DME	RVO vs All	DME vs All
1	Ent	OE_{ent}	.076	.175	**.009**	.251	.754	.117
1	RBP	OE_{RB}	**.009**	.465	**.028**	.175	.917	.175
1	Cos	OE_{ent}	.117	.117	**.009**	.347	.465	.117
1	Cos	OE_{RB}	**.028**	.175	.117	.465	.602	.465
2	Ent	OE_{ent}	**.009**	.251	.076	**.047**	.175	.251
2	RBP	OE_{RB}	**.009**	**.047**	**.016**	**.047**	.754	.076
2	Cos	OE_{ent}	**.028**	.602	.251	**.028**	.602	.251
2	Cos	OE_{RB}	**.009**	.175	**.016**	**.016**	.602	**.028**
4	Ent	OE_{ent}	**.009**	**.009**	**.009**	**.028**	.917	**.016**
4	RBP	OE_{RB}	**.009**	**.016**	**.009**	.251	.465	.117
4	Cos	OE_{ent}	**.009**	**.009**	**.009**	**.047**	.465	**.016**
4	Cos	OE_{RB}	**.009**	.117	**.047**	.117	.175	.602
8	Ent	OE_{ent}	**.009**	**.009**	**.009**	**.009**	.754	**.028**
8	RBP	OE_{RB}	**.009**	**.009**	**.009**	.117	.602	**.028**
8	Cos	OE_{ent}	**.009**	**.009**	**.009**	**.009**	.251	**.016**
8	Cos	OE_{RB}	**.009**	**.009**	**.009**	**.016**	.251	.117
16	Ent	OE_{ent}	**.009**	**.009**	**.009**	**.009**	.754	**.009**
16	RBP	OE_{RB}	**.009**	**.009**	**.009**	**.009**	.117	**.009**
16	Cos	OE_{ent}	**.009**	**.016**	**.009**	.076	.175	**.028**
16	Cos	OE_{RB}	**.009**	**.009**	**.009**	**.047**	**.009**	.347

Table 18. Detection of RVO: statistical analysis (p-value) via two-sided Wilkson ranked sum test accouting for differences in the number of exposed volumes. Bold indicates p-value < .05. RBP: Reject Bucket Probability; Cos: Cosine; Ent: Entropy; Vols: Number of volumes; S: OOD score; M: OOD detection method ; Starg.: stargardt; EC: Exposed class; N/A: No exposure. * indicates cases where comparison was performed with training with 0 volumes using Entropy OOD score.

EC	S	M	0 vs 1	0 vs 2	0 vs 4	0 vs 8	0 vs 16	1 vs 2	1 vs 4	1 vs 8	1 vs 16	2 vs 4	2 vs 8	2 vs 16	4 vs 8	4 vs 16	8 vs 16
Starg.	Ent	OE_{ent}	.142	.540	.111	.391	.066	.251	.754	.917	.602	.465	.175	.117	.602	.754	.602
Starg.	RBP	OE_{RB}	**.002***	**.003***	**.002***	**.002***	**.002***	.917	.347	.602	.602	.465	.602	.754	.465	.917	.754
Starg.	Cos	OE_{ent}	**.007**	.624	.178	.142	.358	.076	.076	.602	.175	.251	.251	.251	.917	.917	.602
Starg.	Cos	OE_{RB}	.391	.221	**.027**	**.037**	**.007**	.465	.076	.251	.175	.347	.917	.602	.465	.347	.465
RVO	Ent	OE_{ent}	.066	**.002**	**.002**	**.002**	**.002**	.251	.076	**.009**	**.009**	.347	**.009**	**.009**	**.016**	**.009**	.347
RVO	RBP	OE_{RB}	**.002***	**.002***	**.002***	**.002***	**.002***	.076	**.016**	**.009**	**.009**	.251	**.009**	**.009**	**.028**	**.009**	.347
RVO	Cos	OE_{ent}	.111	**.003**	**.003**	**.002**	**.002**	.175	.076	**.009**	**.009**	.251	**.016**	**.009**	.117	.076	.676
RVO	Cos	OE_{RB}	**.003**	**.002**	**.002**	**.002**	**.002**	.076	**.016**	**.009**	**.009**	.117	**.009**	**.009**	**.009**	**.009**	.076
DME	Ent	OE_{ent}	.391	.111	**.002**	**.002**	**.002**	.602	**.047**	**.016**	**.009**	.076	**.028**	**.009**	.117	**.016**	.117
DME	RBP	OE_{RB}	**.003***	**.002***	**.002***	**.002***	**.002***	.347	**.028**	**.009**	**.009**	.117	**.009**	**.009**	.175	.076	.175
DME	Cos	OE_{ent}	.391	.142	**.007**	**.005**	**.003**	.602	.175	**.047**	**.016**	.347	.076	**.028**	.347	.076	.117
DME	Cos	OE_{RB}	**.007**	**.020**	**.003**	**.002**	**.002**	.917	.117	**.028**	**.009**	.175	**.047**	**.009**	.251	.076	.251
All	Ent	OE_{ent}	**.005**	**.027**	**.002**	**.002**	**.002**	.754	.076	**.028**	**.009**	**.028**	**.009**	**.009**	.175	**.009**	.347
All	RBP	OE_{RB}	**.002***	**.002***	**.002***	**.002***	**.002***	.076	**.009**	**.009**	**.009**	**.016**	**.009**	**.009**	**.016**	**.009**	**.047**
All	Cos	OE_{ent}	**.005**	.111	**.002**	**.002**	**.002**	.754	.251	.175	**.028**	.117	.076	**.009**	.917	**.028**	**.047**
All	Cos	OE_{RB}	**.037**	**.002**	**.002**	**.002**	**.002**	.465	.347	**.016**	**.009**	.602	**.009**	**.009**	**.016**	**.009**	.602

1.4 Statistical tests - Near-OOD detection

Table 19. ODD scoring metric *Entropy*: statistical analysis (p-value) via two-sided Wilkson ranked sum test for the different ODD detection methods. Bold indicates p-value < .05.

Method	Dirichlet	Dirichlet+OE_{ent}	Ensemble	MCDropout	ODIN	Softmax	Softmax+OE_{RB}	Softmax+OE_{ent}	TTAug	TempScal
Dirichlet	-	**< .001**	**< .001**	**< .001**	**< .001**	**< .001**	**< .001**	**< .001**	**< .001**	**< .001**
Dirichlet+OE_{ent}	**< .001**	-	**< .001**	**< .001**	**< .001**	**< .001**	**< .001**	**< .001**	**< .001**	**< .001**
Ensemble	**< .001**	**< .001**	-	**.009**	**< .001**	**< .001**	**< .001**	**< .001**	.223	**< .001**
MCDropout	**< .001**	**< .001**	**.009**	-	**< .001**	**.002**	**< .001**	**< .001**	.100	**< .001**
ODIN	**< .001**	**< .001**	**< .001**	**< .001**	-	**< .001**	**< .001**	.261	**< .001**	**< .001**
Softmax	**< .001**	**< .001**	**< .001**	**.002**	**< .001**	-	**< .001**	**< .001**	**< .001**	**< .001**
Softmax+OE_{RB}	**< .001**	**< .001**	**< .001**	**< .001**	**< .001**	**< .001**	-	**< .001**	**< .001**	**< .001**
Softmax+OE_{ent}	**< .001**	**< .001**	**< .001**	**< .001**	.261	**< .001**	**< .001**	-	**< .001**	**< .001**
TTAug	**< .001**	**< .001**	.223	.100	**< .001**	**< .001**	**< .001**	**< .001**	-	**< .001**
TempScal	**< .001**	**< .001**	**< .001**	**< .001**	**< .001**	**< .001**	**< .001**	**< .001**	**< .001**	-

Table 20. ODD scoring metric *Cosine*: statistical analysis (p-value) via two-sided Wilkson ranked sum test for the different ODD detection methods. Bold indicates p-value < .05.

Method	Dirichlet	Dirichlet+OE_{ent}	Ensemble	MCDropout	ODIN	Softmax	Softmax+OE_{RB}	Softmax+OE_{ent}	TTAug	TempScal
Dirichlet	-	.069	-	-	**< .001**	**< .001**	**< .001**	**< .001**	-	**< .001**
Dirichlet+OE_{ent}	.069	-	-	-	**< .001**	**< .001**	**< .001**	**< .001**	-	**< .001**
Ensemble	-	-	-	-	-	-	-	-	-	-
MCDropout	-	-	-	-	-	-	-	-	-	-
ODIN	**< .001**	**< .001**	-	-	-	**.005**	**< .001**	**< .001**	-	.076
Softmax	**< .001**	**< .001**	-	-	**.005**	-	**< .001**	**< .001**	-	.253
Softmax+OE_{RB}	**< .001**	**< .001**	-	-	**< .001**	**< .001**	-	**.004**	-	**< .001**
Softmax+OE_{ent}	**< .001**	**< .001**	-	-	**< .001**	**< .001**	**.004**	-	-	**< .001**
TTAug	-	-	-	-	-	-	-	-	-	-
TempScal	**< .001**	**< .001**	-	-	.076	.253	**< .001**	**< .001**	-	-

Table 21. ODD detection method *Softmax*: statistical analysis (p-value) via two-sided Wilkson ranked sum test for the different ODD scoring metrics. Bold indicates p-value < .05.

Metric	Cosine	Dirichlet uncertainty	Entropy	Mahalanobis distance	Maximum probability	RBP	UH	US
Cosine	-	-	-	-	-	-	-	-
Dirichlet uncertainty	-	-	-	-	-	-	-	-
Entropy	**< .001**	-	-	.692	-	-	-	-
Mahalanobis distance	**< .001**	-	-	-	-	-	-	-
Maximum probability	**< .001**	-	**< .001**	**< .001**	-	-	-	-
RBP	-	-	-	-	-	-	-	-
UH	-	-	-	-	-	-	-	-
US	-	-	-	-	-	-	-	-

Table 22. ODD detection method *Softmax+OE_{ent}*: statistical analysis (p-value) via two-sided Wilkson ranked sum test for the different ODD scoring metrics. Bold indicates p-value < .05.

Metric	Cosine	Dirichlet uncertainty	Entropy	Mahalanobis distance	Maximum probability	RBP	UH	US
Cosine	-	-	-	-	-	-	-	-
Dirichlet uncertainty	-	-	-	-	-	-	-	-
Entropy	**< .001**	-	-	**< .001**	-	-	-	-
Mahalanobis distance	**< .001**	-	-	-	-	-	-	-
Maximum probability	**< .001**	-	**< .001**	**< .001**	-	-	-	-
RBP	-	-	-	-	-	-	-	-
UH	-	-	-	-	-	-	-	-
US	-	-	-	-	-	-	-	-
